# Repurposing of Rutan showed effective treatment for COVID-19 disease

**DOI:** 10.3389/fmed.2023.1310129

**Published:** 2023-11-29

**Authors:** Shavkat I. Salikhov, Ibrokhim Y. Abdurakhmonov, Yuliya I. Oshchepkova, Jamolitdin F. Ziyavitdinov, Nodir Sh. Berdiev, Haji Akber Aisa, Jingshan Shen, Yechun Xu, H. Eric Xu, Xiangrui Jiang, Leike Zhang, Natalia L. Vypova, Dilshod Sh. Allaberganov, Nigora A. Tagayalieva, Erkin I. Musabaev, Gulnara A. Ibadova, Ilxom B. Rajabov, Lyubov M. Lokteva

**Affiliations:** ^1^A.S. Sadykov Institute of Bioorganic Chemistry (UzAS), Tashkent, Uzbekistan; ^2^Center of Genomics and Bioinformatics, Academy of Sciences of Uzbekistan, Tashkent, Uzbekistan; ^3^Xinjiang Technical Institute of Physics and Chemistry, Chinese Academy of Sciences, Ürümqi, China; ^4^State Key Laboratory of Drug Research, CAS Key Laboratory of Receptor Research, Shanghai Institute of Materia Medica, Chinese Academy of Sciences, Shanghai, China; ^5^Wuhan Institute of Virology, Chinese Academy of Sciences, Wuhan, China; ^6^Scientific-Research Institute of Virology of the Ministry of the Health of Uzbekistan, Tashkent, Uzbekistan; ^7^Centre for the Development of Professional Qualification of Medical Workers, Tashkent, Uzbekistan

**Keywords:** COVID-19, SARS-CoV-2, 3CLpro, RdRp, *Rhus coriaria*, Rutan, clinical trial

## Abstract

Previously, from the tannic sumac plant (*Rhus coriaria*), we developed the Rutan 25 mg oral drug tablets with antiviral activity against influenza A and B viruses, adenoviruses, paramyxoviruses, herpes virus, and cytomegalovirus. Here, our re-purposing study demonstrated that Rutan at 25, 50, and 100 mg/kg provided a very effective and safe treatment for COVID-19 infection, simultaneously inhibiting two vital enzyme systems of the SARS-CoV-2 virus: 3C-like proteinase (3CLpro) and RNA-dependent RNA polymerase (RdRp). There was no drug accumulation in experimental animals’ organs and tissues. A clinical study demonstrated a statistically significant decrease in the C-reactive protein and a reduction of the viremia period. In patients receiving Rutan 25 mg (children) and 100 mg (adults), the frequency of post-COVID-19 manifestations was significantly less than in the control groups not treated with Rutan tablets. Rutan, having antiviral activity, can provide safe treatment and prevention of COVID-19 in adults and children.

**Clinical Trial Registration:**

ClinicalTrials.gov, ID NCT05862883.

## 1 Introduction

The global coronavirus disease (COVID-19) pandemic has caused an unprecedented problem for public health and socio-economic life (1–3). The virus alters the innate cellular defense using many proteins, resulting in delayed hyper-inflammation and an attenuated interferon (IFN) response (4, 5). When immune activation is delayed, it prolongs the infection and contributes to the long-term consequences of the disease. Along with its vital role in releasing viral proteins, 3C-like proteinase (3CLpro) cleaves specific host proteins and enhances viral replication (6). Inhibition of 3CLpro stops viral replication. It significantly reduces the spread and activity of pathogenic viral proteins responsible for inflammation as well as complications after COVID-19 (7). For many viruses, the protease enzyme plays a critical role in viral protein maturation by purifying pro-proteins after they have been translated into the host cell’s cytosol. As a result, viral proteases are often potential drug targets (8, 9). Many antiviral drugs have been developed against viral infections by targeting proteases. For example, HIV-1 protease inhibitors (Tipranavir, Darunavir, Amprenavir, Lopinavir, Saquinavir, Atazanavir, Indinavir, Ritonavir, and Nelfinavir) (3) and hepatitis C virus (HCV) NS3/4A protease inhibitors (Boceprevir, Telaprevir, Ritonavir, Asunaprevir, Paritaprevir, Grazoprevir, Glecaprevir, Voxilaprevir, and Sofosbuvir) (2) are FDA-approved drugs.

The absence of a human 3CLpro homolog makes this protein one of the most attractive targets for antiviral drugs (10). In addition, 3CLpro is highly conserved among corona-viruses, and by summarizing the structural similarities between viruses, 3CLpro may facilitate the development of broad-spectrum antivirals. Significant advances were made in late 2021 with the introduction of Nirmatrelvir (also known as PF-07321332), in combination with Ritonavir, developed by Pfizer and named “Paxlovid” (11). Through effective reversible covalent inhibition of 3CLpro, Nirmatrelvir-based therapy has been shown to reduce the risk of severe complications following COVID-19 infection in unvaccinated, hospitalized symptomatic adults by 89% (12).

RNA-dependent RNA polymerase (RdRp) plays an essential role in the RNA virus life cycle and has no homologs in the host cell (13, 14). This opens up opportunities for developing antiviral drugs and reduces the risk of protein damage in human cells. Generally, viral RdRPs are considered low-fidelity enzymes mainly due to the lack of proofreading functions (15). Nucleoside analogs target the functionally and structurally conserved coronavirus polymerase, and their insertion into viral RNA causes premature termination or a lethal increase in mutations (16).

An analysis of existing and developing drugs shows that developing 3CLpro-targeted medications and drugs that inhibit RNA-dependent RNA polymerase against SARS-CoV-2 should be valuable for the long-term fight against this constantly evolving viral pathogen (17). Due to minimal treatment options for SARS-CoV-2 and its newly emerging chimeric variants, there is an urgent need for novel and effective oral antiviral drugs with both protease and RNA polymerase inhibition activities to combat COVID-19 to reduce the consequences of post-COVID health issues (18). It plays an essential role in managing patients with COVID-19, particularly in reducing the risk of hospitalization and death from COVID-19 infection.

In the Republic of Uzbekistan, earlier studies have established the effectiveness of *Rhus coriaria*—derived Rutan 25 mg against strains of influenza A and B viruses, adenoviruses, paramyxoviruses, herpes virus, and cytomegalovirus (19). Rutan has anti-radical antioxidant activity and increases the resistance of living organisms to conditions characterized by oxygen deficiency. Additionally, Rutan has a stabilizing effect on cardiomyocytes, thereby preserving the vital activity of myocardial cells. On experimental models of hemic and normobaric hypoxia, it was shown that the antihypoxant activity of Rutan was more effective than that of quercetin and mexidol, which are used in medical practice. This phenomenon can make a significant contribution to the compensation of oxidative stress caused by coronavirus infection and to the therapeutic effect of the drug.

The result obtained suggests the use of Rutan as an antiviral drug with pronounced antihypoxic properties to eliminate respiratory dysfunction, heart failure, circulatory disorders, and other pathological conditions that occur during coronavirus infection.

Based on the results of clinical trials obtained from the three clinical hospitals of the Republic of Uzbekistan in 2015, the Ministry of Health of the Republic of Uzbekistan recommended Rutan as an antiviral drug for treating influenza viruses. Because of the importance of repurposing and using existing antiviral molecules against COVID-19 viral infection (20), we investigated the effectiveness of Rutan tablets against SARS-CoV-2. Here, we presented the results of repurposing research of Rutan in the laboratory *in vitro* experiments and in preclinical as well as in randomized, open-label, controlled clinical studies to determine its effectiveness against SARS-CoV-2 as an antiviral drug with multiple components for the treatment of coronavirus infection.

## 2 Materials and methods

### 2.1 Isolation of polyphenols

The collected leaves of tannic sumac were dried in air at room temperature without direct sunlight. The dried raw materials were crushed by hand. Ground plant material (250 g) was extracted twice with 40% ethanol in a ratio of 1:10 for 8 h. The resulting extracts were filtered and distilled in a rotary evaporator to remove alcohol, and then the aqueous portion was extracted three times with ethyl acetate. The obtained extracts were distilled in a rotary evaporator, and chloroform was added to the residue in a ratio of 1:3 by volume (v:v) to precipitate the polyphenols. The process was carried out for 4 h until the precipitate was formed entirely. The resulting precipitate was separated, washed in 20 mL chloroform, and air-dried. The precipitation yield was 15%.

### 2.2 Isolation of pure substances

Individual substances were isolated in HPLC Agilent Technologies 1200 (Agilent Technologies, Santa Clara, CA, United States). The semi-preparative column, XSelect CSH Prep C18, 5 μm, 10 × 250 mm (Waters, Massachusetts, United States), was used for chromatography. The Phenomenex C18, 5 μm, 4.6 × 250 mm (Waters, Massachusetts, United States), was used for analytical analyses. A 0.1% solution of trifluoroacetic acid was used as solution A, and acetonitrile was used as solution B: acetonitrile gradient: 0 min—15%, 28 min—25%, 33–38 min—60%, 43 min—15%. The flow rate was 3 mL/min in both cases. In all cases, a UV detector with a wavelength of 269 nm was used (19).

### 2.3 Mass spectral analysis of purified substances

The mass spectral analysis of the obtained substances was further studied on an Agilent Technologies 6520B Q-TOF LC–MS (Agilent Technologies, Santa Clara, CA, United States); the ionization source was ESI; dryer gas flow—5 L/min (300°С); skimmer tone—20 V; fragment-125 V; the range of mass detection in MS mode is 100–2000 m/s and in target mode of MS/MS is 50–2000 m/s, collision energy—35, 50 eV; the ionization method is negative. Samples were subjected to mass spectrometry with an Agilent Technologies 1200 series HPLC: Zorbax SBC18 column (3 μm, 0.5 × 150 mm). The composition of the mobile phase: A—0.1% formic acid, B—acetonitrile +0.1% formic acid. The elution rate was 15 μL/min. A gradient of solution B was performed: 5 min—20%, 20 min—25%, 25 min—30%, 30 min—60%, and 35 min—20%. The solution was passed through an Agilent Technologies 1260 μ degasser. Samples were loaded onto the column at a 0.1 mg/mL concentration via Agilent Technologies Micro WPS (21).

### 2.4 RNA elongation inhibition assay

A 5′-FAM labeled RNA duplex (with the template sequence 5′-UUUUUUUUUUAUAACU-UAAUCUCACAUAGC-3′ and the primer sequence 5′-FAM-GCUAUGUGAGAUUAAGUUAU-3′) was prepared as previously described (22), as well as the recombinant SARS-CoV-2 RdRp used in this work. To begin the assay, a 4X reaction buffer (4XRB) containing 80 mM Tris (pH 8.0), 40 mM KCl, 24 mM MgCl_2_, and 0.04% Triton-X100 was prepared using DEPC-treated water. SARS-CoV-2 RdRp was diluted to 4 μM, and the RNA duplex was diluted to 12 μM in 4XRB. Rutan and its isolates were dissolved in PBS to a concentration of 20 μM, 100 μM, and 500 μM, respectively, for use. For each reaction, 4.5 μL RdRp (4 μM), 4.5 μL RNA duplex (12 μM), and 9 μL Rutan or Rutan isolates at different concentrations were added. The mixture was incubated at room temperature in the dark for 1 h. Then, 2 μL ATP (100 mM) was added to each reaction, and the mixture was placed in a water bath at 37°C for 1 h. Finally, 40 μL quench buffer (94% formamide, 30 mM EDTA, prepared with DEPC-treated water) was added to stop the reaction. A 10 μL reaction product sample was mixed with 2 μL of 6X DNA loading buffer (Thermo Scientific). An example of 10 μL was loaded onto a 15% urea-PAGE denaturing gel. The gels were run at 150 V for 90 min and imaged with a Bio-Rad imager.

### 2.5 RNA-dependent RNA polymerase inhibition activity

The reaction was carried out in a black 96-well plate with a flat bottom. The reaction mixture contained 20 mM Tris-HCl, pH 8.0, 10 mM KCl, 6 mM MgCl2, 500 μM ATP, 20 μg/mL poly-U, 0.1 mg/mL BSA, and 0.25 μM SYTO 9 (50 μM stock solution in TE buffer, pH 7.5). The polymerization reaction was initiated by adding 250 nm SARS-CoV-2 RdRp, and fluorescence was recorded for 30 min at 30°C. To determine the *K*_m_ and *V*_max_ constants for SARS-CoV-2 RdRp binding to poly-U RNA, standard reactions were performed at increasing matrix concentrations (0.5–50 μg/mL) in the presence of 500 μM ATP. Kinetic parameters for ATP were obtained from assays in the presence of increasing concentrations of this nucleotide (200–2,250 μM) and using 3 μg/mL poly-U. IC_50_ values were obtained from standard reactions in the presence of 3 μg/mL poly-U and 1,500 μM ATP and increasing concentrations of each inhibitor. The reaction was stopped after 60 min by adding 25 mM EDTA to the samples. Negative control was run in parallel to determine the level of background fluorescence, with the reaction stopped before the addition of SARS-CoV-2 RdRp.

### 2.6 Cell lines and viruses

African green monkey kidney Vero E6 cells (ATCC-1586) were maintained in Dulbecco’s modified Eagle’s medium (DMEM) with 10% fetal bovine serum (FBS) and 1% penicillin and streptomycin antibiotics. Cells were kept at 37°C in a 5% CO_2_ atmosphere. The strains of 2019-nCoV-WIV04 were obtained from the Microorganisms & Viruses Culture Collection Centre and were propagated in Vero E6 cells. All experiments with authentic SARS-CoV-2 viruses were conducted in the Biosafety Level 3 facility of the Wuhan Institute of Virology, Chinese Academy of Sciences (CAS), China.

### 2.7 Anti-SARS-CoV-2 activity assay

In our study, Vero E6 cells were pre-seeded to 48-well plates (50,000 cells/well) for 16–18 h and treated with a medium containing a gradient concentration of Rutan at 100 μL/well for 1 h to investigate an antiviral effect of Rutan through the entry phase of viral propagation. The cells were inoculated with SARS-CoV-2 at a multiplicity of infection (MOI) of 0.01. As the 1 h action is not much impacted by the volume of the medium, 1 h later, the supernatant was removed, and cells were washed with PBS and treated with fresh medium containing a gradient concentration of Rutan at 200 μL/well. At 24 h post-infection, the cell supernatant was collected. Antiviral activities were evaluated by quantifying viral copy numbers in the cell supernatant via real-time fluorescence quantitative PCR (qRT-PCR), as described in our previous study (23). The inhibition rate was calculated based on the viral copy number, and the 50% effective concentration (EC_50_) was calculated with GraphPad Prism software 8.0. At least three independent experiments were performed.

### 2.8 3CLpro protease inhibition activity

A fluorescence resonance energy transfer (FRET) protease assay was applied to measure the inhibitory activity of compounds against the SARS-CoV-2 3CLpro. The fluorogenic substrate (Dacyl-KTSAVLQSGFRKME-Edans) was synthesized by GenScript (Nanjing, China). The assay was performed in a total volume of 120 μL. In brief, the recombinant SARS-CoV-2 3CLpro at a final concentration of 50 nM was mixed with serial dilutions of each compound in 80 μL of assay buffer (50 mM Tris-HCl, pH 7.3, 1 mM EDTA) and incubated for 10 min at ambient temperature. The reaction was initiated by adding 40 μL of a fluorogenic substrate with a final concentration of 10 μM. After that, the fluorescence signal at 340 nm (excitation)/490 nm (emission) was measured immediately every 35 s for 3.5 min with a Bio-Tek Synergy-H1 plate reader. The initiated velocities of reactions with compounds added at various concentrations compared to those added with DMSO were calculated and used to generate inhibition profiles. IC_50_ measurements were carried out for each compound in three biological replicates for each data point. These data were used to calculate the IC_50_ value via nonlinear regression analysis using GraphPad Prism software 8.0 (GraphPad Software, Inc., San Diego, CA, United States).

### 2.9 Preclinical studies of the substance of the drug Rutan

The studies were carried out based on international and national regulatory documents (24–26) by qualified staff on 5–6 weeks-old CD-1 mice (both sexes, weighing 18–20 g, *n* = 170); 12 weeks old adult Wistar Hannover rats (both sexes, weighing 180–200 g, *n* = 235), 3–4 weeks old developing Wistar Hannover rats (both sexes, weighing 25–30 g, *n* = 60), 24–28 weeks old Hartley strain guinea pigs (both sexes, weighing 300–350 g, *n* = 24), and 18–20 weeks old Сhinchilla rabbits (both sexes, weighing 2.5–3 kg, *n* = 18). According to conducted tests, the type of animals, group sizes, doses, types of administration of Rutan, and duration of experiments are presented in Supplementary Tables S1, S2.

The animals were kept in standard vivarium conditions (humidity: 55%–65%, temperature: 22°C ± 2°C) with free access to drinking water and laboratory food. All manipulations with the animals complied with the European Directive 2010/63/EU on protecting animals used for scientific purposes (24). The protocol was approved by the Animal Ethical Committee based on the Institute of Bioorganic Chemistry, AS RUz (Protocol Number: 133/1a/h, dated August 4, 2014). All surgery was performed under sodium pentobarbital anesthesia, and all efforts were made to minimize suffering. Preclinical tests in developing rats (general toxicity) were additionally conducted with the possible use of Rutan in pediatric practice (27). The acute oral toxicity of Rutan was performed by fixed dose procedure (25). During experiments, animal health and behavior were monitored daily. The following criteria for a pain assessment were considered: fractures, self-induced trauma, abnormal vocalization, abnormal posture or movements, open wounds or ulcers, and others (28). In the case of detection in experimental animals of specific endpoint criteria (29), they were immediately euthanized.

### 2.10 Clinical studies of Rutan in adults

The ongoing study was a randomized, open-label, controlled study to evaluate the safety and efficacy of the drug, which was designed and conducted by the Scientific Research Institute of Virology, Ministry of Health of the Republic of Uzbekistan, Tashkent, Uzbekistan.

Participants were recruited from November 23, 2020, to February 16, 2021. The randomized trial design and eligibility criteria for participants and their recruitment for the trials were performed by the Scientific Research Institute of Virology, Ministry of Health of the Republic of Uzbekistan, Tashkent, Uzbekistan (detailed in Supplementary Files S1–S3). The control group received treatment according to the National Temporary Protocol No. 7 for COVID-19, and the main group received additional Rutan 100 mg two times daily for 10 days. The analysis of laboratory parameters was carried out upon admission by the criteria for inclusion in the comparison groups. All ongoing and related trials for this drug/intervention in adults have been registered at ClinicalTrials.gov with ID NCT05859919. The reason for not registering the clinical trials in adults and children (see below) before the study is that there is no requirement for clinical trial registration in international registries before the study in our country’s regulations.

The main parameters studied during the tests were the determination of C-reactive protein (CRP), PCR analysis of a swab from the nasopharynx for coronavirus infection, interleukin-6 (IL6), D-dimer at admission and on the 5th day of treatment, reflecting mainly the processes of inflammation in the organism and their dynamic change during treatment.

### 2.11 Clinical studies of Rutan in children

A randomized, open-label, controlled clinical trial was designed and conducted by the Scientific Research Institute of Virology, Ministry of Health of the Republic of Uzbekistan, Tashkent, Uzbekistan. Participants were recruited from the period of July 2021 to June 2022. the Scientific Research Institute of Virology, Ministry of Health of the Republic of Uzbekistan, Tashkent, Uzbekistan. The control group received treatment according to the National Temporary Protocol No. 7 for COVID-19, and the main group received additional Rutan 25 mg 2 times a day for 10 days. Patients were monitored daily with an analysis of the dynamics of clinical symptoms until the patients were discharged from the hospital. The examination was carried out according to a specially developed patient examination card, with the clinical investigation results recorded in the patient’s medical history and the examination card. Laboratory studies included several biochemical tests, including determining the level of CRP, procalcitonin, D-dimer, ferritin, and IL6.

Virological studies included detecting the SARS-CoV-2 virus in nasal secretion by PCR upon admission of patients and in dynamics every 24 h after the first collection until a negative result and discharge of patients. Clinical examination of patients (follow-up study) was carried out within 4 to 6 months after the discharge of patients from the hospital to identify possible post-COVID-19 conditions. All ongoing and related trials for this drug/intervention in children have been registered at ClinicalTrials.gov with ID NCT05862883.

### 2.12 Statistical analysis

In pharmaco-toxicological studies of pre-clinical trials with the normally distributed data in groups, the parameters were presented as the mean (*M*) and mean error (*m*), and an unpaired *t*-test was used to compare the means of studied groups.

The Kolmogorov–Smirnov test and the Shapiro–Wilk test were used to assess the normality of quantitative indicators. With a non-normal data distribution, we utilized medians and 25% and 75% quartiles for subsequent analysis (clinical studies in adults and children). Additionally, the chi-square test was used for categorical data from clinical studies to compare differences in symptoms and disease stages between the two groups. A *p*-value less than *p* < 0.05 was considered statistically significant. Statistical calculations were performed using SPSS 26.0 (Illinois, Chicago, United States).

### 2.13 Ethics approval of the research

For a human study, the approval of the Pharmacological Ethics Committee of the Republic of Uzbekistan (No. 6/13-1456, Protocol No. 6, dated October 30, 2020). The Institute of Bioorganic Chemistry’s ethics board (Uzbekistan) reviewed and approved the relevant protocols for the study (Supplementary Files S1–S3). All methods were performed in accordance with the relevant guidelines and regulations. We used the CONSORT reporting guidelines for the randomized, open-label, controlled clinical trials in adults and children (30).

After recruiting participants, according to the form of the disease, written consent was taken to participate in a clinical trial in adults, and written consent was obtained from the next of kin of children to participate in a clinical trial. The developed questionnaire was filled out. Randomization was carried out with inclusion in one of the two study groups using basic therapy or basic therapy with the inclusion of the Rutan. The sample sizes were determined based on consent and questionnaire outcomes.

All the experiments were carried out in accordance with the relevant guidelines and regulations. Participants’ IDs were renumbered and deidentified so no one, even researchers, could know the identity of the patients. The authors had no information to identify individual participants during or after data collection.

## 3 Results and discussions

### 3.1 Isolation and antiviral activity of Rutan

From the dried leaves of tannic sumac *Rhus coriaria*, growing on the territory of the Republic of Uzbekistan, following successive extraction with 40% ethyl alcohol and ethyl acetate, followed by precipitation of polyphenols, the sum of polyphenols was obtained, which is used later to develop the drug Rutan, containing five main components in the ratio: 39.2: 14.8: 26.3: 8.4: 6.7. The structures of the compounds were established based on extensive spectroscopic analysis (1D and 2D NMR and MS) and identified (Table 1).

**Table 1:** Characteristics of the main components/fractions of the drug Rutan.

ID	Mw	Structure	Description
R-5	940	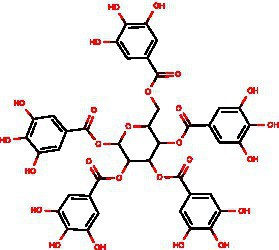	1,2,3,4,6-penta-O-galloyl-β-D-glucose
R-6	1,092	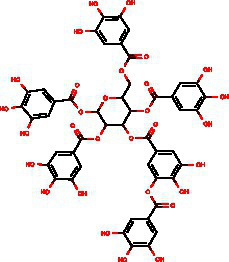	3-bis-O-galloyl-1,2,4,6-tetra-O-galloyl-β-D-glucose
R-7	1,244	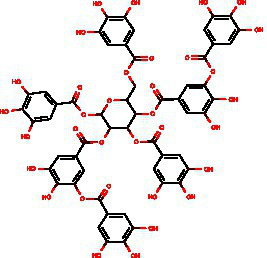	2,4-bis-O-galloyl-1,3,6-tri-O-galloyl-β-D-glucose
R-8	1,396	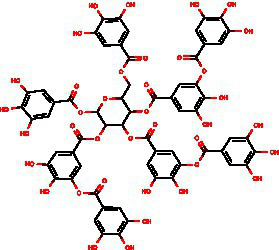	2,3,4-bis-O-galloyl-1,6-di-O-galloyl-β-D-glucose
R-9	1,548	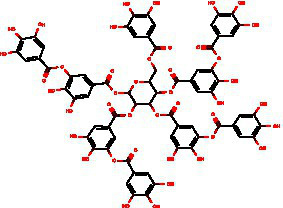	1,2,3,4-bis-O-galloyl-6-O-galloyl-β-D-glucose

**Figure 1:**
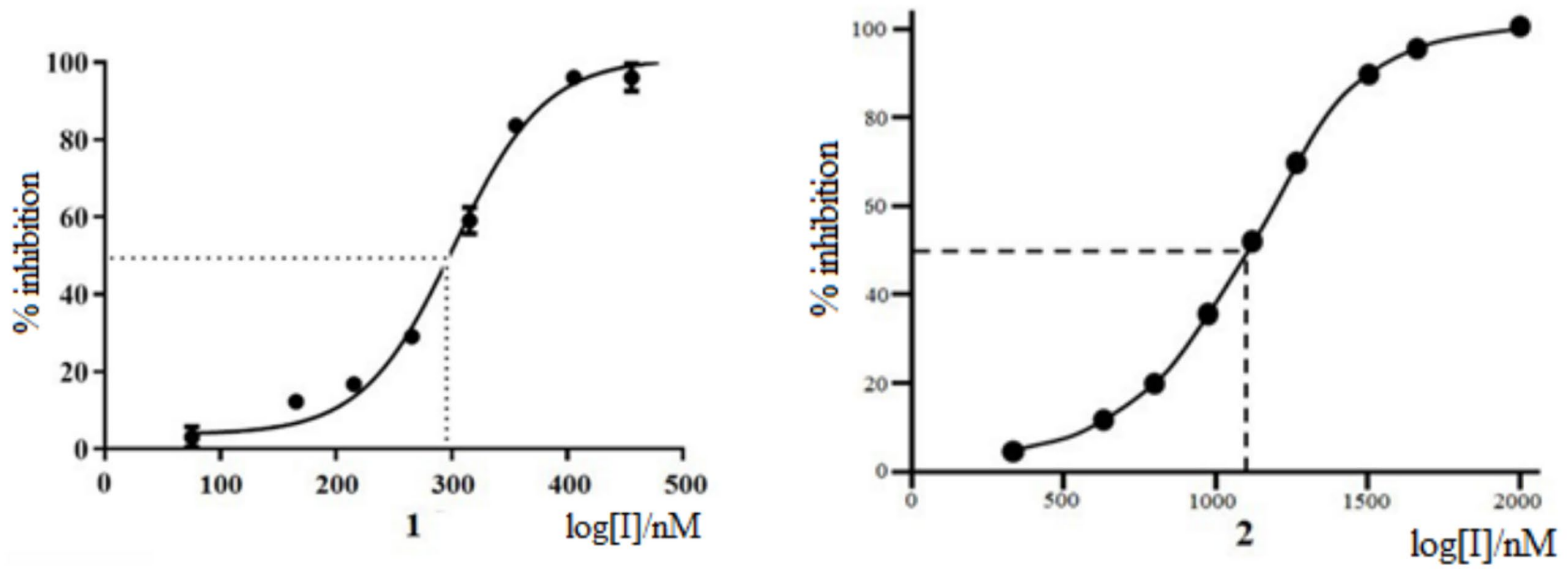
SARS-CoV-2 RdRp inhibition by Rutan (1) and Myricetin (2).

The antiviral activity of Rutan against influenza A/California/07/09 (H1N1) strain was tested on MDCK cells compared with Favipiravir (31–33) as a positive control. The experiments showed that Rutan’s EC_50_ (effective 50% concentration) was 3.2 μM. At the same time, the EC_50_ of Favipiravir under the same conditions was 24 μM (Supplementary Figure S1). The results showed a relatively low level of cytotoxicity in the concentration of test compounds required to reduce cell viability by 50% (CC_50_). The concentration range was just over 50 μM. The selectivity index (SI) of Rutan was over 15. We determined that Myricetin inhibits SARS-CoV-2 RdRp at an IC_50_ concentration of 1,100 nM, as shown in Figure 1.

At the same time, Rutan has inhibitory activity against SARS-CoV-2 RdRp at an IC_50_ concentration of 291 nM, inhibiting SARS-CoV-2 RdRp of SARS-CoV-2 is 3.8 times more effective than Myricetin (33). The results show that the drug Rutan exhibits inhibitory activity against RdRp and may be promising as a drug that prevents the replication and multiplication of the virus in the cell.

### 3.2 RdRp inhibitory activity of Rutan

**Figure 2:**
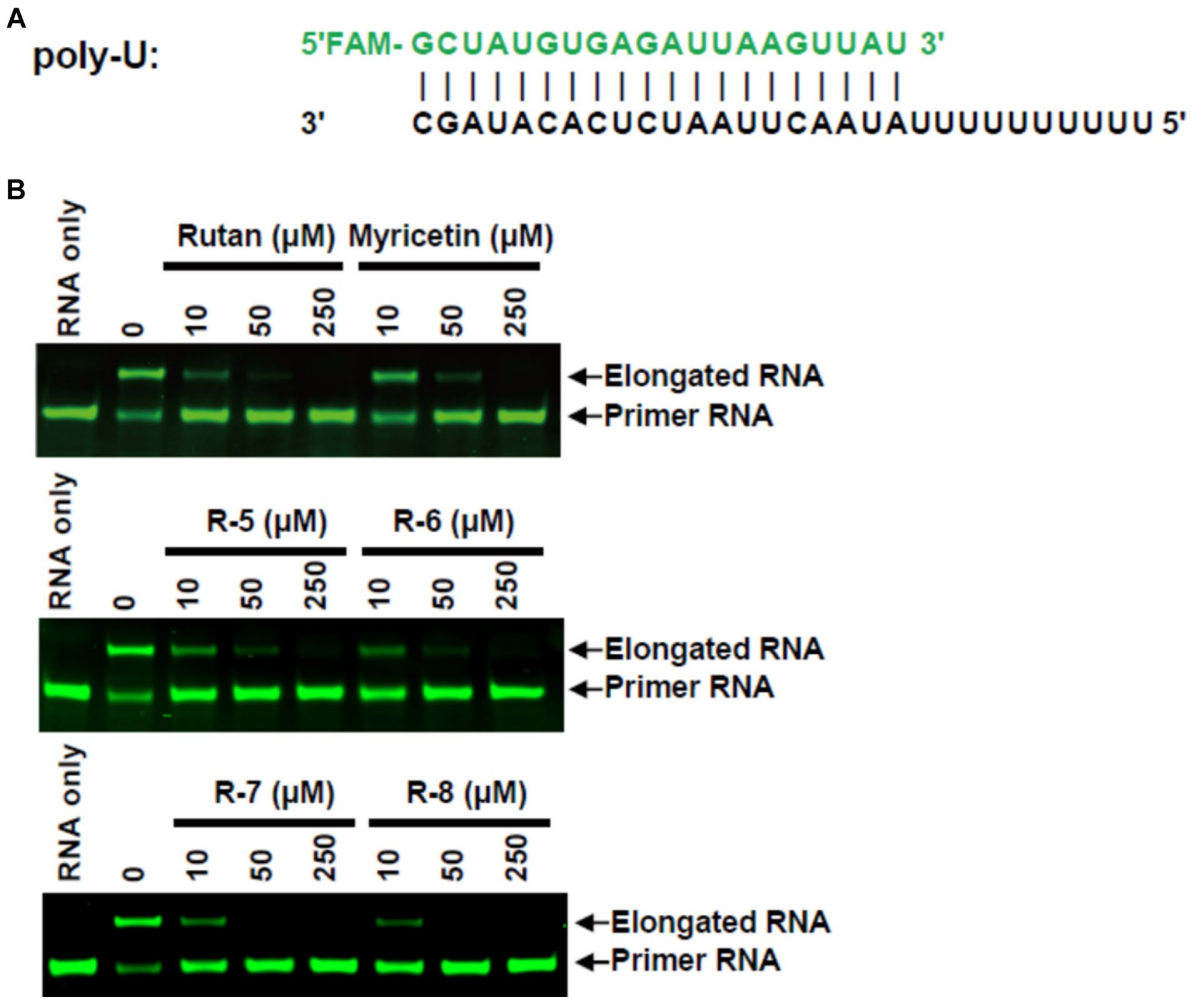
RdRp inhibition by Rutan and myricetin **(A)** RNA duplex sequence with U 10 5′-overhang as a template for primer extension and assembly of the RdRp-RNA complex. **(B)** Gel assays for partial RNA duplex elongation by purified RdRp complex and its inhibition by Rutan and its main components and myricetin.

Further study of the mechanism of RdRp inhibitory activity of Rutan showed that it blocks the formation of the RdRp-RNA complex, preventing efficient binding of SARS-CoV-2 RdRp to RNA with the participation of two accessory proteins nsp7 and nsp8 and subsequent RNA polymerization. In an *in vitro* experiment where the nsp12-nsp7-nsp8 complex showed RNA polymerization activity on a poly-U template upon adding adenosine triphosphate (ATP), the active triphosphate form of Remdesivir inhibited polymer elongation (22). In our experiment, adding 10–50 μM Rutan and its main components resulted in partial or complete inhibition of poly-U primer elongation. Myricetin showed inhibitory activity in higher concentrations. The addition of 250 μM Rutan and Myricetin completely blocked the formation of the RdRp-RNA complex (Figure 2).

The results presented in Figure 2B show the presence of synergism in the inhibitory activity of the main components of Rutan, which manifests itself in an increase in SARS-CoV-2 RdRp inhibition by the sum. The results show that the drug Rutan exhibits inhibitory activity against RNA-dependent RNA polymerase and may be promising as a drug that prevents the replication and multiplication of the virus in the cell.

**Figure 3:**
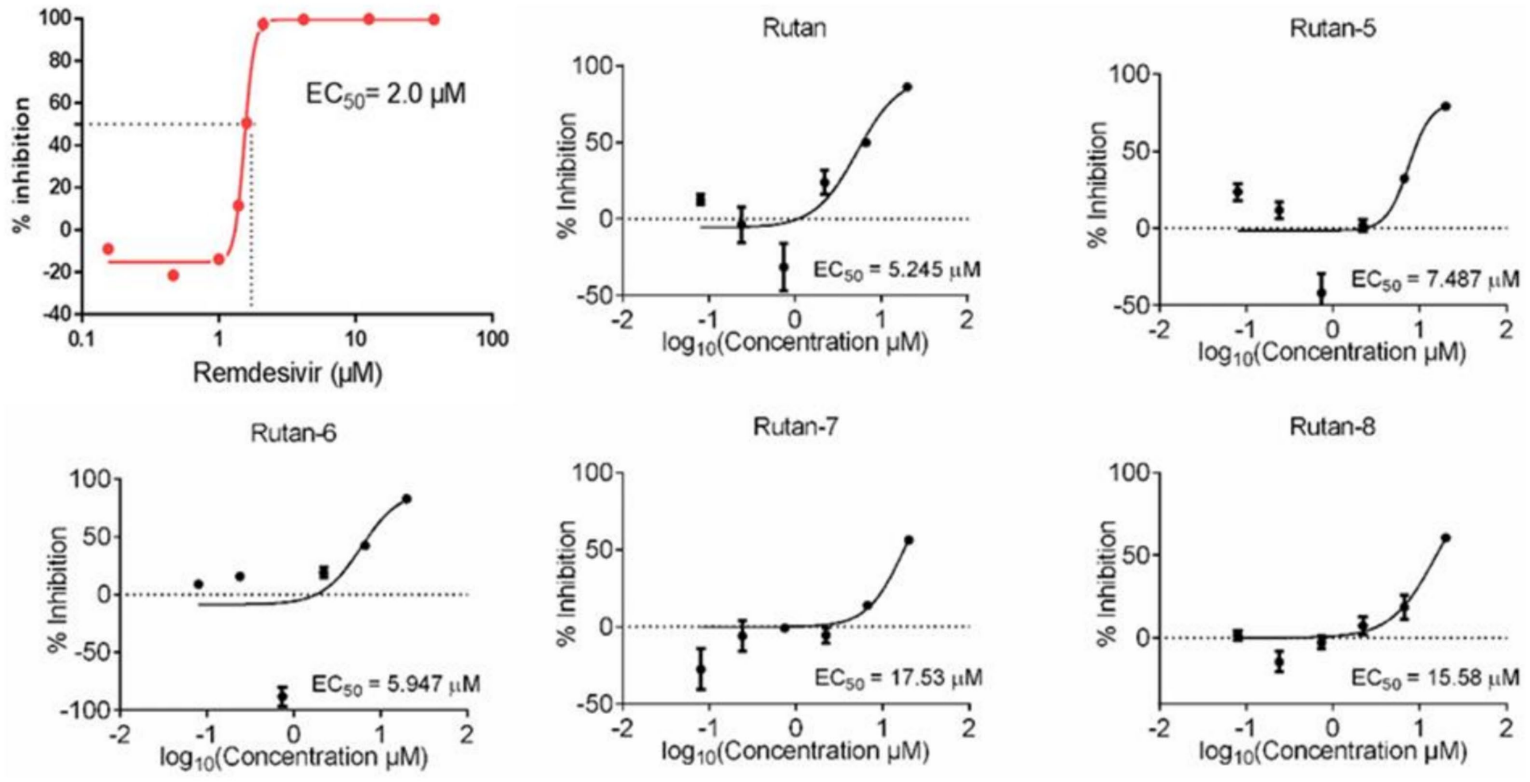
Remdesivir, Rutan and components activity against SARS-CoV-2 virus in Vero E-6 cells.

In addition, on a culture of Vero E6 cells infected with SARS-CoV-2, the viability of uninfected host cells after exposure to Rutan for 72 h was measured on a counter to identify and exclude compounds with a cytotoxic effect. The reference drug was Remdesivir. The results presented in Figure 3 showed the presence of synergism in the inhibitory activity of the main components of Rutan, which manifests itself in increased inhibition of the SARS-CoV-2 virus in total. The results showed that the practical EC_50_ value for Rutan appears at a concentration of 5.245 μM. Remdesivir in the same cells reaches a sufficient concentration of EC_50_ at a dose of 2.0 μM. A comparative study of the antiviral activity of Rutan with Remdesivir showed that the effective antiviral concentration EC_50_ of Rutan against the SARS-CoV-2 virus is 2.6 times less than that of Remdesivir. However, Remdesivir has numerous side effects (34, 35). The South Korean Centres for Disease Control and Prevention (CDC) has acknowledged that Remdesivir cannot reduce the spread of the virus in the early stages of the disease, as it is only used for hospitalized patients with severe illness.

### 3.3 3CLpro protease inhibition activity

Due to the many severe consequences and complications after COVID-19, the most studied and crucial drug target for the treatment of COVID-19 is the 3CLpro, given its indispensability in the viral maturation and replication cycle. Numerous studies have shown a positive effect of small molecular compounds in inhibiting 3CLpro SARS-CoV-2 (36). When studying the dependence of Rutan in the range from 0.5 μM to 50 μM in the laboratory *in vitro* experiments on the inhibition of 3CLpro enzymatic activity, it was determined that Rutan has antiviral activity, inhibiting 3CLpro of the SARS-CoV-2 virus at a concentration of 0.5 μM by 17.7%. With an increase in the concentration of Rutan to 50 μM, a dose-dependent increase in its inhibitory activity occurred up to 97.5%, which indicates the possibility of significant suppression of virus reproduction *in vivo* (Table 2).

**Table 2:** Dependence of the effectiveness of inhibition of the enzyme 3CL protease of the SARS-CoV-2 virus on the concentration of Rutan.

Name	3CLpro inhibition, %		
	0.5 μmol	5 μmol	50 μmol
Rutan	17.7	64.4	97.5

**Figure 4:**
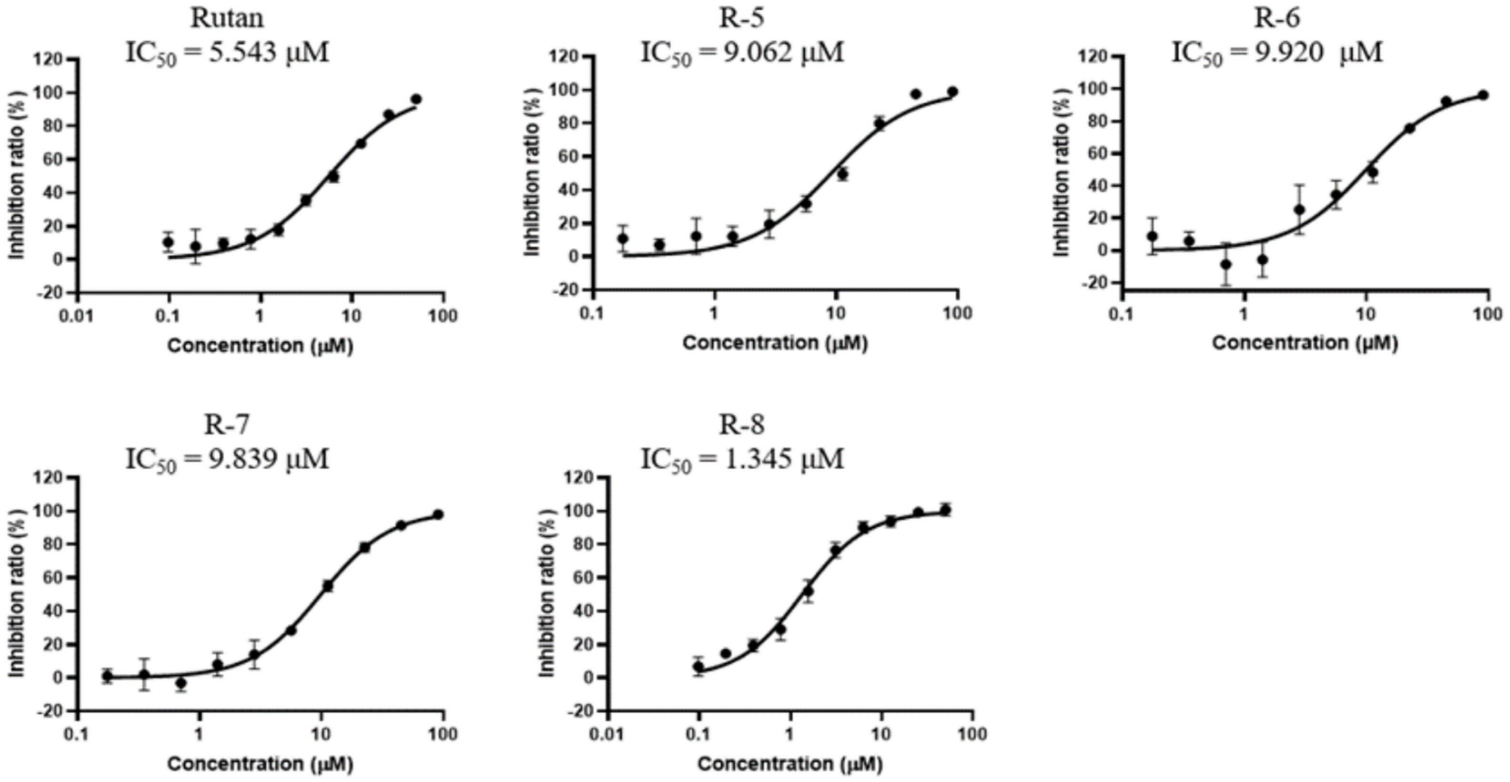
Inhibition activity of Rutan and components on SARS-CoV-2 3CLpro.

A natural product, Baicalein (5,6,7-trihydroxy flavone), was chosen as a reference drug in our study, originally isolated from the roots of *Scutellaria baicalensis* and *Scutellaria lateriflora*, a promising antiviral drug for the treatment of COVID-19 (37). In another study, clinical trials conducted in China showed that Baicalein was well tolerated in treating acute or chronic hepatitis (38). A comparative study of the inhibitory activity of Rutan and the flavonoid Baicalein showed that the IC_50_ against 3CLpro-SARS-CoV-2 was 2.9 μM and 0.94 μM for Rutan and Baicalein, respectively (Supplementary Figure S2). Because the drug Rutan is a sum of ellagitannins with a proven structure, additional studies were carried out on the inhibitory activity of its main components. The substrate of recombinant SARS-CoV-2 3CLpro was used as a target in the experiments. The results presented in Figure 4 show the presence of the potential synergism in the inhibitory activity of the main components of Rutan/ This requires further studies, which reveal an understanding of increased inhibition of the main protease (Mpro) of SARS-CoV-2 in total.

Previously reported IC_50_ values for coronavirus 3CLpro inhibition for Boceprevir, an inhibitor of hepatitis C virus protease (approved for use by the FDA), are 4.5 μM. Lopinavir, a proven coronavirus 3CLpro inhibitor, has an IC value of 234 μM (39), and quercetin, previously identified as the most potent inhibitor of SARS-CoV-2 3CLpro among 150 different compounds, inhibits the protease at an IC_50_ concentration of 23.4 μM with an inhibition constant (*K*_i_) 7.4 Μm (40).

Our results show that the Rutan substance and its main components exhibit a higher percentage of inhibition of the main coronavirus protease, with IC_50_ values of 1.3–9.9 μM (Figure 4), which indicates the prospect of further research for their use as an antiviral drug for the treatment of COVID-19. Antiviral activity studies have shown that Rutan, represented by the sum of polyphenols from *Rhus coriaria*, effectively inhibits two vital enzyme systems of the SARS-CoV-2 virus: 3CLpro and RdRp of the virus and can be used as an antiviral medicine for the treatment of coronavirus infection.

### 3.4 Toxicity and immunomodulatory studies in animals

Intragastrical LD_50_ of Rutan on mice (outbred ICR CD-1) was in the range of more than 5,000 mg/kg (Category 6 in the GHS); on rats, it was in the range of 2,000–5,000 mg/kg (Category 5 in the GHS) with no cumulative properties.

During the chronic toxicity study, intragastrical administration of Rutan to rats at the doses of 25, 50, and 100 mg/kg/day during 30 days did not result in any significant changes in the general condition of animals, hematological and biochemical parameters, urinalysis, systematic anatomy, organ weight, or histopathological examination in the treatment groups compared with the control group. Rutan does not cause irreversible changes, genotoxicity, immuno-toxicity, reproductive toxicity, local irritation, or allergic reactions (Supplementary Table S1).

The immunomodulatory properties of Rutan were manifested by humoral immunity activation (the pronounced increase in the number of antibody-producing cells), enhancement of nonspecific resistance (the increase of phagocytic activity of neutrophils), the increase in the cellularity of the thymus, lymph nodes, bone marrow, simultaneously with the decrease in the development of delayed-type hypersensitivity (Supplementary Table S1).

Additional toxicity testing on young rats was conducted according to the regulatory guidelines (25). These studies confirmed the immunomodulation effect of Rutan: the hyperplasia of immune organs T- and B-zones and MALT structures were revealed after 30-day intragastrical administration of Rutan at the doses of 10, 20, and 40 mg/kg/day to young rats (Supplementary Table S2 and Supplementary Figure S3). Thus, the studies have shown that high doses of Rutan are relatively safe for rats and mice due to the absence of any drug accumulation in the organs and tissues of experimental animals or abnormal results of acute and chronic toxicity found after its use.

### 3.5 Clinical studies in adults

The results made it possible to conduct randomized, open-label, controlled clinical trials of Rutan to identify the possibility of recommending the drug for clinical use in the Republic of Uzbekistan in patients with SARS-CoV-2 coronavirus infection by the current laws of the Republic of Uzbekistan and the ethical principles of the Declaration of Helsinki. The study included 121 patients of both sexes older than 18 years. According to the randomization of CONSORT guidelines (29), patients were divided into four groups depending on the severity of the disease.

**Figure 5:**
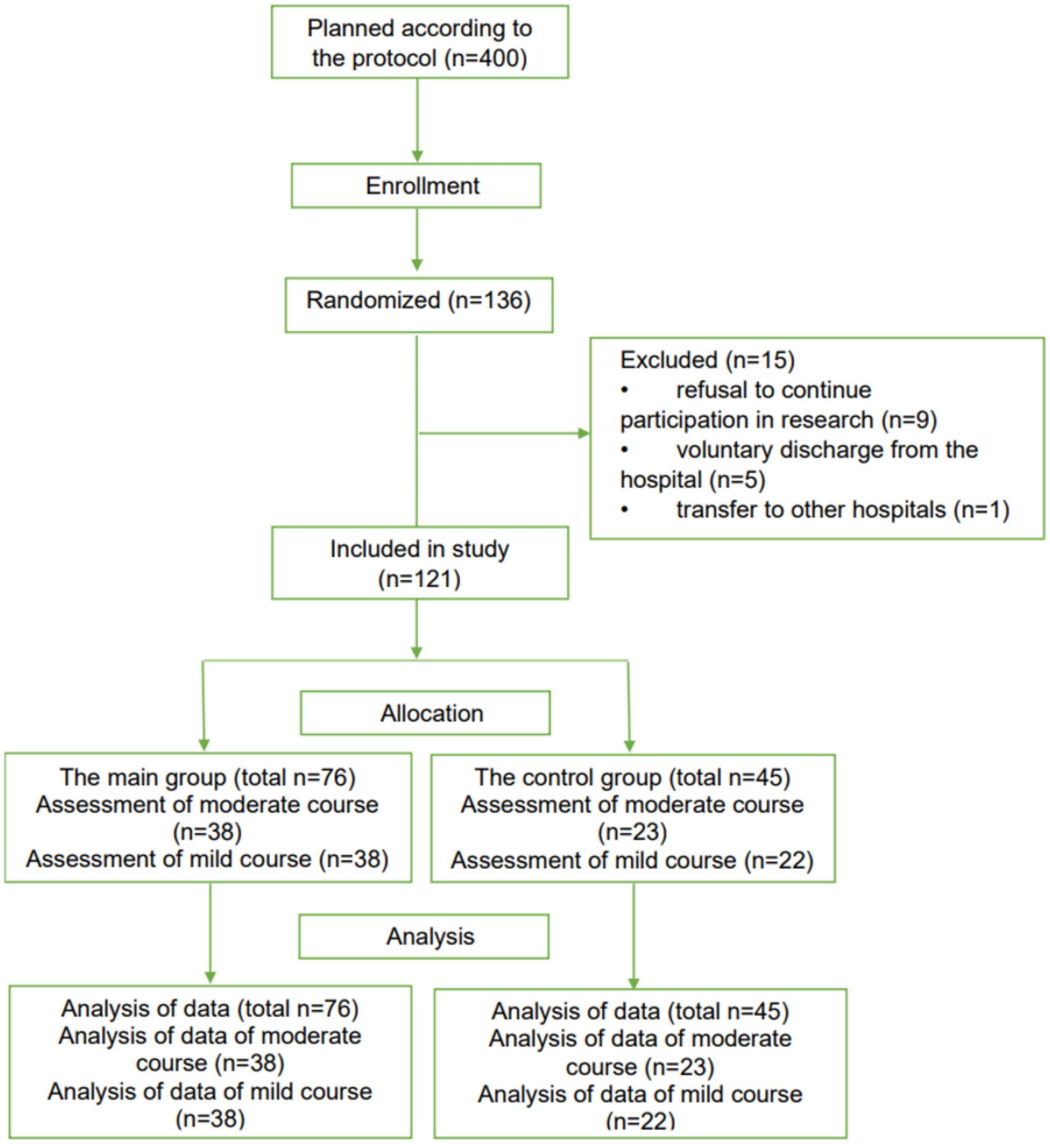
The CONSORT-based block diagram on randomized clinical trial results in adults.

The 61 PCR-positive patients (*n* = 61) with a moderate course of the disease with parenchymal damage up to 30% (38 patients in the main group and 23 patients in the control group) and 60 PCR-positive patients with a mild course of the disease (38 patients in the main group and 22 patients in control group) were included in the study (Figure 5).

The main groups of patients received Rutan 100 mg tablets twice daily for 10 days. Control groups received therapy according to the National Interim Protocol 7 for managing patients with coronavirus infection COVID-19. Mild COVID-19 disease was defined as mild illness (with or without respiratory symptoms) with oxygen saturation >96%. The moderate course of the disease was described as a disease (with damage to the lung parenchyma up to 30%) with an oxygen saturation of 94%. Mild illness included symptoms of an uncomplicated viral upper respiratory tract infection such as chills, weakness, lethargy, dizziness, cough (with or without sputum production), agility, anosmia, muscle pain, sore throat, dyspnoea, nasal congestion, or headache. In rare cases, patients also experienced diarrhea, nausea, and vomiting (Supplementary Table S3). The moderate form was additionally accompanied by shortness of breath, an increased level of shortness of breath (Supplementary Table S4).

According to the results of a study of patients of the main and control groups with the mild and moderate course of the disease, an analysis of several clinical symptoms revealed the significant effect of Rutan. The results showed that in the groups of patients treated with Rutan, weakness, lethargy, headache, dizziness, sore throat, anosmia, ageusia, and myalgia decreased much earlier from individual COVID-19 symptoms and were absent at the discharge of patients (Supplementary Tables S3, S4). On average, the patient’s discharge occurred on day seven after taking Rutan. During treatment with Rutan, patients did not show an association between the underlying disease and severe side effects, and no patient stopped taking Rutan due to side effects.

In addition, as a result of treatment with Rutan 100 mg, two times a day, in the experimental group of patients with a mild course of the disease on the 5th day of hospitalization, PCR analysis of a swab from the nasopharynx for coronavirus infection COVID-19 was negative in 79% of patients. At discharge, the percentage of patients with negative PCR was 100%. At the same time, in the control group, PCR analysis of a swab from the nasopharynx on day 5 was negative only by 14.6%, remaining positive in 54.5% of patients at the time of discharge (Supplementary Table S5). PCR analysis of a nasopharyngeal swab for coronavirus infection COVID-19 in the main group of patients with a moderate course of the disease in 84.2% of patients on day 5 was negative, amounting to 100% at discharge. This figure was 9 and 61.8% in the control group, respectively (Supplementary Table S6). To determine the severity of the inflammatory syndrome in patients, all patients at admission and on the 5th day of hospitalization underwent laboratory tests, which are the most informative according to the protocol for managing patients with COVID-19, including the determination of (CRP) C-reactive protein, PCR analysis of a smear from nasopharynx for coronavirus infection, interleukin-6 (IL6), D-dimer, reflecting mainly the processes of inflammation in the body and their dynamic change during treatment.

During infectious or inflammatory diseases, CRP levels can activate the classical complement cascade of the immune system and modulate the activity of phagocytic cells, maintaining the role of CRP in the opsonization of infectious agents and dead or dying cells (41). The level of CRP at the admission of patients is a sensitive and early indicator of the severity of COVID-19 (42), which correlates with lung damage and the severity of the disease. Compared to other standard laboratory parameters, CRP levels are significantly associated with the severity of COVID-19. The study of CRP in patients revealed a significant decrease in the index in the main group on the 5th day after taking the drug in 70% of patients (*p* < 0.02) compared with the control group. None of the patients required oxygenation during treatment. A significant reduction in CRP associated with a decrease in inflammation in patients with COVID-19 treated with Rutan may benefit the post-acute consequences of COVID-19. Thus, “Rutan Tablets 0.1 g” No. 20 has been approved for widespread use in the treatment of COVID-19 of moderate severity and with lung damage up to 30%, as well as against all strains of influenza for adults.

### 3.6 Clinical studies in children

Although children and adolescents with COVID-19 appear less susceptible and have milder symptoms after infection, they are also at risk of developing severe stages (43). It is known that children and adults have physiological differences (44); accordingly, many effective COVID-19 drugs for adults are unsuitable for children. The basis for studying Rutan in children was the lack of direct antiviral medicines for COVID-19.

The herbal antiviral medicine “Rutan, tablets 25 mg,” which has previously shown its effectiveness in children with influenza and other acute respiratory viral infections (22), was used to treat children and adolescents aged 6–18 years in the complex therapy of COVID-19 with a mild and moderate course of the disease in a randomized, open-label, controlled clinical trials, following CONSORT guidelines (30).

**Figure 6:**
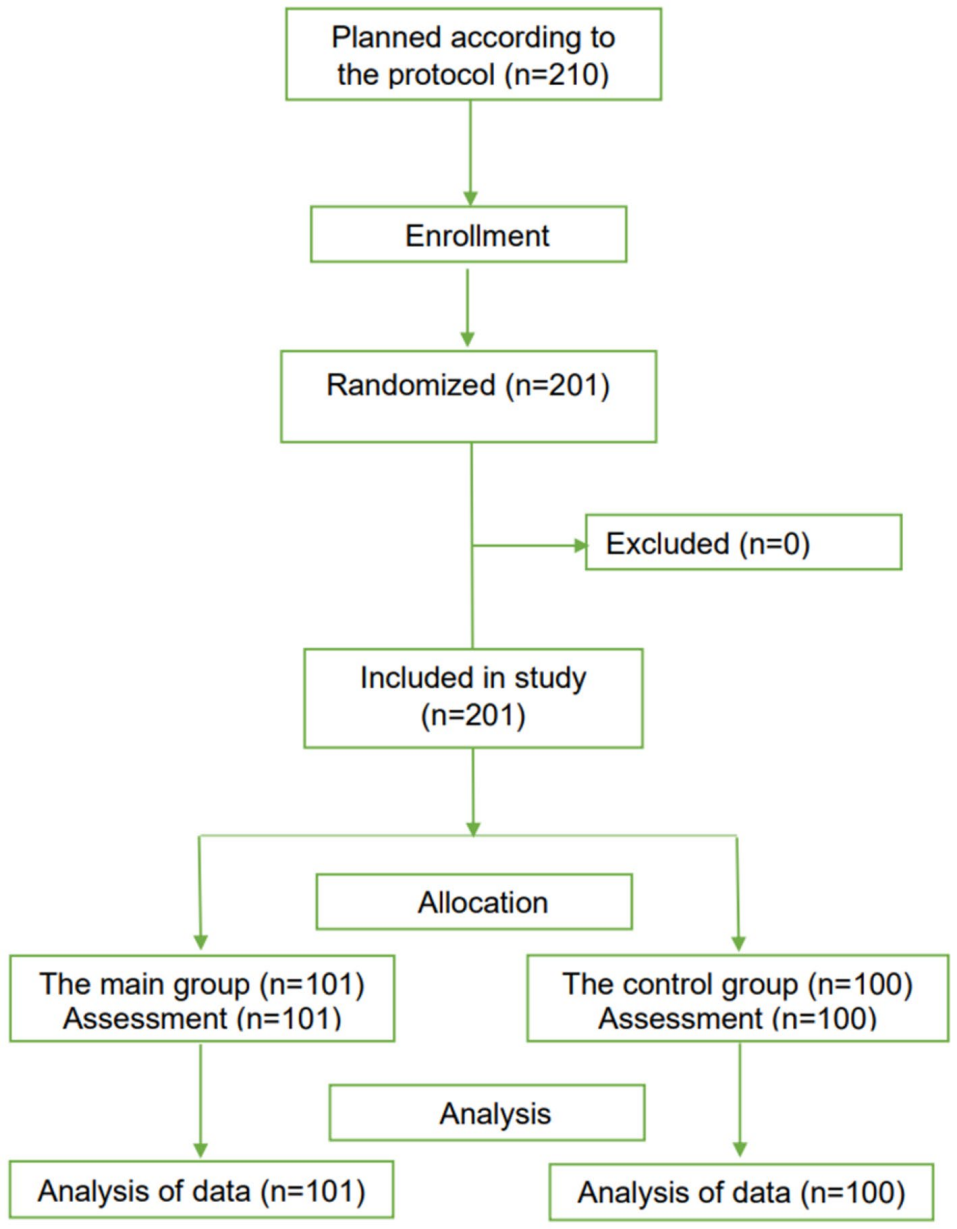
The CONSORT-based block diagram on the results of a randomized clinical trial in children.

Two hundred one PCR-positive children aged 6 to 18 years (101 patients in the main group and 100 patients in the control group) were included in the study (Figure 6).

An analysis of the clinical manifestations of the disease in children and adolescents showed the polymorphism of lesions in COVID-19. The most frequently observed symptoms at admission were fever from sub-fibril to fibril values. During our study, it was found that the drug Rutan at a dose of 25 mg in children aged 6–18 years does not have side effects when used in the complex therapy of children with COVID-19, does not have antagonistic effects when used together with the drugs specified in the treatment protocols this disease in children.

According to the results of the study of patients of the main and control groups, an analysis of several clinical symptoms of the disease in patients of the compared groups revealed the significant effect of Rutan 25 mg. This was especially noticeable in such indicators as the relief of hyperthermia, a decrease in the severity of cough, shortness of breath, restoration of appetite, weakness, lethargy, and other manifestations of the disease compared with the control group of patients (Supplementary Table S7). It was noted that in patients with a mild course of the disease, clinical symptoms stopped within 3–5 days of hospital stay, and the severity of symptoms decreased by 2–3 days. In the moderate course, the duration of symptoms persisted up to 5–7 days of hospital stay, and the severity of symptoms decreased by 3–4 days of treatment.

As in the adult group, the dynamics of biochemical indicators of inflammation, such as C-reactive protein and D-dimer, were studied in the children’s group. In contrast to adults, children did not show a statistically significant increase in these parameters at the height of the disease before using Rutan 25 mg and in the stage of convalescence in the experimental group. A similar pattern was observed in the control. The results obtained showed that the use of the drug Rutan 25 mg in the complex therapy of children aged 6–18 years with COVID-19 is characterized by the absence of side effects, good tolerance, and harmlessness for the organism of this group of patients and has a pronounced clinical and significant antiviral effect in patients with a mild and moderate course of the disease. Analysis of the dynamics of the virus in nasal secretion in patients aged 6–18 showed significant differences in patients of the comparison groups. So, according to the results of the virus study on the 5th day of sampling in the group of children who received Rutan, the virus was practically undetected. In contrast, in the control group who did not receive this drug, the indicated indicator was four times higher than that in the main group (*p* < 0.05).

In connection with the emerging long-term symptoms observed after the disease in adults and children, possible post-COVID conditions were analyzed in the examined children after discharge from the hospital within 4 to 6 months. The questionnaire was compiled based on the disease symptoms that prevailed during the illness and was supplemented with the most frequently recorded post-COVID manifestations described in the literature. In the main group, there were 62 patients; in the control group, 65 patients, respectively. According to the results of the study, it was found that in patients who received Rutan in the complex therapy of patients, in 13 cases, there were several clinical symptoms of the disease, such as prolonged weakness, fatigue, acute respiratory viral infections up to 1 month after suffering COVID-19.

Among patients in the control group who did not receive Rutan, 26 patients with post-COVID symptoms were noted, which was two times more than among patients who received Rutan 25 mg twice a day in complex therapy (*p* < 0.05). Also, it should be noted that patients in the control group had not only a high frequency of post-COVID conditions and symptoms but also their greater severity and variety: weakness, lethargy, frequent acute respiratory viral infections, abdominal pain, prolonged cough, fatigue, decreased appetite, changes in taste preferences, loss of taste and smell, etc. (Supplementary Figure S4).

Based on the presented data, the manifestations of asthenia-vegetative (14 patients of the main group—22.6% and 20 children of the control group—30.8%, respectively) and inflammatory-pain syndrome (4 children from the main group—6.4% and nine children from the control group—13.8%, respectively) were evident in patients of the compared groups of patients. The frequent detection of catarrhal respiratory syndrome was detected in 5 patients of the main (8.1%) and five children (7.7%) of the control group (*p* > 0.05). Analysis of the frequency of post-COVID conditions in children depending on gender did not show significant differences in boys and girls (*p* > 0.05).

An analysis of the frequency of detection of post-COVID conditions in different age groups showed that in the main group of patients, post-COVID conditions were more often recorded in patients of the adolescent group 15–18 years old, in the group of children 6–10 and 11–14 years old, the frequency of post-COVID conditions was less pronounced (Supplementary Table S8). In the control group of children who did not receive Rutan, the incidence of post-COVID symptoms was most often recorded in the group of children aged 6–10 and 15–18 years; in the group 11–14 years, these symptoms were less common. This gives grounds to recommend introducing Rutan as part of the basic means for treating COVID-19 in children with mild and moderate diseases. There is a need to study the synergetic effects and cellular mechanisms of action of Rutan fractions analyzed in this study. The additional limitation of the study, however, could be the medium size of the participants involved in our single-institution clinical trials in the Uzbekistan population. Future large-scale, multi-national/multi-institutional clinical trials should be conducted, which require international cooperation and additional investment.

## 4 Conclusion

Our studies have shown that Rutan effectively inhibits two vital enzyme systems of the SARS-COV-2 virus: 3CL protease and RNA-dependent RNA polymerase, combining the properties of combination drugs and, therefore, the synergism of action *in vivo* conditions enhancing the anti-COVID-19 effect. Our preclinical studies have shown that Rutan when used long-term at doses of 25, 50, and 100 mg/kg, is safe due to the absence of any accumulation of the drug in the organs and tissues of experimental animals. No abnormal results of acute and chronic toxicity were detected after its use. Additionally, Rutan was shown to have immunostimulating activity for humoral, cellular, and innate immunity. The combination of all proven biological activities in one drug that has passed preclinical and randomized, open-label, controlled clinical trials involving adults and children makes Rutan a promising, safe medicine for combating COVID-19 infection and its complications in monotherapy of mild and moderate forms of the disease, as well as in combination with more effective anti-COVID-19 drugs in severe conditions of the course of the disease.

## Data availability statement

The original contributions presented in the study are included in the article/Supplementary material, further inquiries can be directed to the corresponding author.

## Ethics statement

The studies involving humans were approved by the Pharmacological Ethics Committee of the Republic of Uzbekistan. The studies were conducted in accordance with the local legislation and institutional requirements. Written informed consent for participation in this study was provided by the participants’ legal guardians/next of kin. The animal study was approved by the Pharmacological Ethics Committee of the Republic of Uzbekistan. The study was conducted in accordance with the local legislation and institutional requirements.

## Author contributions

SS: Conceptualization, Funding acquisition, Supervision, Writing – original draft, Writing – review & editing. IA: Formal analysis, Methodology, Supervision, Validation, Writing – original draft, Writing – review & editing. YO: Investigation, Methodology, Project administration, Writing – review & editing. JZ: Formal analysis, Investigation, Methodology, Project administration, Writing – review & editing. NB: Investigation, Visualization, Writing – review & editing. HA: Conceptualization, Funding acquisition, Investigation, Methodology, Supervision, Validation, Writing – review & editing. JS: Conceptualization, Funding acquisition, Investigation, Methodology, Project administration, Validation, Writing – original draft, Writing – review & editing. YX: Conceptualization, Investigation, Methodology, Validation, Visualization, Writing – review & editing. HX: Investigation, Methodology, Validation, Visualization, Writing – review & editing. XJ: Investigation, Methodology, Validation, Visualization, Writing – review & editing. LZ: Conceptualization, Investigation, Methodology, Project administration, Supervision, Validation, Visualization, Writing – original draft, Writing – review & editing. NV: Formal analysis, Investigation, Validation, Visualization, Writing – review & editing. DA: Data curation, Investigation, Visualization, Writing – review & editing. NT: Formal analysis, Investigation, Methodology, Project administration, Writing – review & editing, Data curation. EM: Conceptualization, Data curation, Formal analysis, Funding acquisition, Investigation, Project administration, Resources, Visualization, Writing – original draft, Writing – review & editing. GI: Formal analysis, Investigation, Methodology, Resources, Visualization, Writing – review & editing. IR: Data curation, Formal analysis, Investigation, Validation, Visualization, Writing – review & editing. LL: Data curation, Formal analysis, Methodology, Resources, Validation, Visualization, Writing – review & editing.
