# Supplementary material for: Repurposing of Rutan showed effective treatment for COVID-19 disease
Source: Front Med (Lausanne). 2023 Nov 29;10:1310129. doi: 10.3389/fmed.2023.1310129 (PMC10716264; doi:10.3389/fmed.2023.1310129)
Supplement: Supplementary file 1 [file Data_Sheet_1.docx]

**Protocol**

**Conducting clinical trials to study the effectiveness of the local medicine Rutan 100 mg in adults and Rutan 25 mg in children in the treatment of COVID-19 and Acute Respiratory Virus Infection (ARVI) development of treatment protocols**

**Protocol to:** Salikhov SI, Abdurakhmonov IY, Oshchepkova YI., et al. Repurposing of Rutan showed effective treatment for COVID-19 disease.

**CONTENT**

COMPLIANCE STATEMENT …….……………………………….………….….…………...5

1 SUMMARY OF THE PROTOCOL

1.1 Overview…………………………………………………….…………….…..……………..5

1.2 Schemе..……………………………………………….…………….…….….……................6

1.3 Schedule of activities. ……. ………………………….…………….…….….……………..8

2 Introduction…………………………………………………………….…….….…….............9

2.1 Rationale for the study ………………………………………………….….…..…………..9

2.2 Background……..…….……………………..……………………………..……………….12

2.3 Risk/Benefit Assessment …………….….…………………………….…….……………..13

2.3.1 Risk/Benefit Assessment ………….…………….….…………………….……………...13

2.3.2 Known potential risks ……………….………….…………….……….………………..14

2.3.3 Assessment of potential risks and benefits ……………………………….…………..14

3 OBJECTIVES AND ENDPOINTS ………………………………………….……………....14

4. STUDY DESIGN …………………………………….……………………….……………...15

4.1 Overall design ..…………………………………………………………….……………...15

4.2 Scientific rationale for the study design …….…………………………….…………….16

4.3 Dose justification ………………………………………………………….……………….17

4.4 Determination of the end of the study .………………………………….………………17

5. STUDY POPULATION ………….…………………………………….…………………...17

5.1 Inclusion criteria …….……………….………………………………….…………………17

5.2 Exclusion criteria …….…….……………………………………………….……………...18

5.3 Lifestyle …………………….…..…………………………………………….…………….18

5.4 Strategies for study acceptance ………………………………………………….……….19

6 STUDY THERAPEUTIC AGENT .............................…………………….…….…………..19

6.1 RUTAN 0.025 and its application ………………………………………………………..19

6.1.1 Description of the therapeutic agent ..…………………………………………………19

6.1.2 Dosage and administration ..……………….…………………………………………..20

6.2 Preparation/processing/storage/reporting...................……………..……….…………..20

6.2.1 Acquisition and Accountability…………………...……………………………………20

6.2.2 Formulation, appearance, packaging and labeling…..........……………….………....20

6.2.3 Product storage and stability…..............……………………………….………………21

6.2.4 Preparation..………………………………………………………….…………………..21

6.3 Error minimization measures: randomization and masking ……..…………………..21

6.4 Research procedures…….…..…………………………………………………………….21

6.5 Accompanying therapy………….………....……………………………………………..21

6.5.1 Medical emergencies……….……………………………………………………………21

7 STUDY TERMINATION AND TERMINATION/PARTICIPANT WITHDRAWAL....21

7.1 Termination of the study……..………...…………………………………………………21

7.2 Termination of participation/participant withdrawal from study……..……...……...22

7.3 Feedback and re-visit issues ……….……………………………………………………..22

8 RESEARCH METHODS AND PROCEDURES………………..……………….…………22

8.1 Performance Evaluation…...…………………………………………………….………...22

8.1.1 NEW assessment table……………………………………………………….………….24

8.2 Study safety and other measures ……………………………….…………………...….25

8.3 Adverse and serious adverse events ………………………………………….….…….25

8.3.1 Definition of adverse event …………………………………………..…….….….25

8.3.2 Definition of serious adverse events .………….………………………………..…25

8.3.3 Adverse event classification ………………….………………………………..….26

8.3.3.1 Severity of event ..........………………………………………………………….26

8.3.3.2 Attitude toward the study …………………………………………….....……….26

8.3.3.3 Expectations …………………………………………………………………..….27

8.3.4 Time and frequency of event assessment and follow-up …….………..……………27

8.3.5 Adverse event reporting .........……………………………….……………………..27

8.3.6 Severe adverse event reporting …………………….……………………………....28

8.3.7 Event reporting to participants ……………………………………..………….…...28

8.3.8 Reporting pregnancy ………………………………………………….………….…28

8.4 Unforeseen problems …………………..……………………………….………….….28

8.4.1 Identifying unexpected problems …………...………………………….…….……...28

8.4.2 Communicating unforeseen problems to participants………………………….…..29

9 STATISTICAL CONSIDERATIONS …………………………………………………..29

9.1 Statistical hypotheses ……………………………………………………..……………29

9.2 Populations for analysis ...................................................................................................29

9.3 Statistical analysis ............................................................................................................29

9.3.1 General approach ..........................................................................................................29

9.3.2 Primary endpoint analysis .............................................................................................29

9.3.3 Secondary endpoint analysis .........................................................................................30

10 ADDITIONAL DOCUMENTATION AND FORMS OF OPERATION …………........30

10.1 Regulatory, ethical, and research oversight rules ...........................................................30

10.1.1 Informed Consent Process ...........................................................................................30

101.1.1 Parent/Guardian Authorization and Child Consent Requirements

(in the case of a minor)……………………………………………………………………...30

10.1.1.2 Other informed consent procedures ..........................................................................31

10.1.2 Termination and closure of the study ..........................................................................31

10.1.3 Confidentiality and privacy ..........................................................................................32

10.1.4 Secondary use of stored samples and data ...................................................................32

10.1.4.1 Data sharing for secondary research .........................................................................32

10.1.5 Key roles and study leadership ....................................................................................32

10.1.6 Safety oversight ............................................................................................................32

10.1.6.1 Research Team Oversight .........................................................................................33

10.1.6.2 Safety Oversight Committee .....................................................................................33

10.1.7 Clinical monitoring .......................................................................................................33

10.1.8 Data processing and record-keeping .............................................................................33

10.1.8.1 Data collection and management responsibility .........................................................33

10.1.8.2 Retention of study records ..........................................................................................34

10.1.8.3 Source records ............................................................................................................34

10.1.9 Deviations from protocol ..............................................................................................34

10.1.10 Publication and Data Exchange Policy .......................................................................34

10.1.11 Publication ..................................................................................................................34

10.2 Abbreviations ...................................................................................................................35

11 References ...........................................................................................................................35

**COMPLIANCE STATEMENT**

The trial will be conducted in accordance with the International Conference on Harmonization of Good Clinical Practice (ICH GCP), Regulations for Clinical Trials at the Research Institute of Virology, Ministry of Health of the Republic of Uzbekistan. The principal investigator guarantees that no deviations or changes in the protocol will take place without the prior consent of the sponsor of the investigational new drug and documented approval of the ethics committee, except when necessary to eliminate the immediate danger to the trial participants. All personnel involved in this study are familiar with the human subject protection material and the ICH GCP.

The protocol, informed consent form(s), recruitment materials, and all participant materials will be sent to the ethics committee for review and approval. Approval of both the protocol and the consent form must be obtained before any participant can enroll. Any amendment to the protocol will require review and approval by the ethics committee before changes can be made to the study. The ethics committee will approve all changes to the consent form; a decision will be made as to whether new consent from the consenting participant must be obtained using the previously approved consent form.

**1 SUMMARY OF THE PROTOCOL**

**1.1 Overview**

**Title:** Study of the effectiveness of the local medicine Rutan 100 mg in adults and Rutan 25 mg in children in the treatment of COVID-19 and Acute Respiratory Virus Infection (ARVI) development of treatment protocols

**Research description:** The Research Institute of Virology of the Ministry of Health of the Republic of Uzbekistan tested the drug Rutan 0.025 on a limited number of coronavirus patients. According to the results obtained, the drug attenuates the virus by 73.7% starting from the 5th day of the disease compared to the control (57.1%). Undoubtedly, these results should be verified in larger clinical trials, and the efficacy of this drug in children from 6 to 18 years old should be confirmed, depending on the severity of the patients, the combined treatment as an antiviral component should be chosen with a thorough analysis of the effectiveness and safety.

**Objectives:**

**Main objective:**

Evaluation of the efficacy and safety of the registered drug "Rutan 0.1", previously used against influenza viruses, against SARS-CoV-2 in adults аnd to determine the efficacy and tolerability of the domestic drug "Rutan tablets 0,025" in children and adolescents 6-18 years old with COVID-19 and/or ARVI

**Optional: 1.** To choose the optimal treatment scheme depending on the patient’s severity. 2. Investigate the possibility of combined treatment of "Rutan 0.1" for adults with drugs prescribed by the recommended current national protocol. 3. To study the possibility of combined treatment of "Rutan 0.025"for children with drugs prescribed by the current recommended national protocol.

**Study Group:**

The study is planned to include 400 patients of both sexes, over 18 years old and 210 patients of both sexes, 8 to 18 years of age with diagnoses: Coronavirus infection caused by COVID-19 (confirmed), mild/moderate form U07.1. Patients are registered on the basis of the document of the Republic of Uzbekistan: Interim recommendations for the management of patients infected with COVID-19 (sixth version) dated August 04, 2020. The severity of the condition will be assessed according to the criteria of the current national protocol for the diagnosis and management of patients with COVID-19 (Appendix No. 1). For comparison, a comparison group with identical properties will be compiled. The main group and the matching group will be separated by randomization.

**Description of the study intervention:**

In this clinical trial on adults, we plan to prescribe Rutan 0.1 on the first day, 1 tablet 3 times a day, in subsequent days, 1 tablet 2 times a day. The duration of the course of treatment is 10 days, with good tolerability of the drug. During the study, dose adjustment is possible.

Duration of observation: 3 months.

In this clinical study on children, we plan to prescribe "Rutan 0.025" 1 tablet 2 times a day. The duration of the course of treatment is 10 days, if the drug is well tolerated. Dose adjustment is possible during the study.

**Duration of observation: 6 months.**

**1.2 Diagram**

| **Preparatory stage** | Randomization  Obtain informed consent. Screening of potential participants against inclusion and exclusion criteria. |
| --- | --- |
|  |  |
| First Visit Initiation of Inpatient/Outpatient Treatment. | Conduct an initial study. General clinical (detailed blood test) and biochemical studies (calcium, sodium, potassium, alkaline phosphatase (ALP), alanine aminotransferase (ALT), aspartate aminotransferase (AST), total blood bilirubin and its fractions, blood urea nitrogen, creatinine, C-reactive protein, ferritin, interleukin-6 (IL-6)) blood. Instrumental research methods (ultrasound examination of the abdominal organs, chest X-ray, multispiral computed tomography (MSCT), electrocardiogram (ECG). Issue an individual card of the patient under study. |
| Second Visit: Daily/every other day during inpatient/outpatient treatment. | Physical examination. Repeat the study at elevated rates (if applicable) |
|  | Physical examination. Repeat the study at elevated rates or with clinical deterioration. |
| Visit Third Daily/Every Other Day during Inpatient/Outpatient Treatment**.** |  |
|  |  |
| Visit Fourth On Day 10 of Inpatient/Outpatient Treatment | Physical examination. Conduct repeated laboratory and biochemical analyzes. Polymerase chain reaction test for coronovirus of a new type (COVID-19). Instrumental data (if necessary). Fill out the questionnaires or refer to section 1.3, Schedule of events. |
| One month after inpatient/outpatient treatment | Assessment of the condition investigated by telephone/registered letter/local equivalent methods. Fill out the questionnaires or refer to section 1.3, Schedule of events.  Assessment of the condition investigated by telephone/registered letter/local equivalent methods. Fill out the questionnaires or refer to section 1.3, Schedule of events. |
| Three months after inpatient/outpatient treatment. | **Final evaluations based on research results** |
|  |  |

**1.3 Schedule of activities**

| **Procedures** | Screening | Screening 1 | Screening 2 | Screening 3 | Screening 4 | After 1 month | After 3 months |
| --- | --- | --- | --- | --- | --- | --- | --- |
| Informed Consent | X |  |  |  |  |  |  |
| Personal data | X |  |  |  |  |  |  |
| Questionnaire | X |  |  |  |  | X | X |
| Randomization | X |  |  |  |  |  |  |
| Necessary range of investigations |  | X | X | X | X |  |  |
| Related therapy | X | X | X | X | X |  |  |
| Physical exam | X | X | X | X | X |  |  |
| Vital signs | X | X | X | X | X |  |  |
| Height | X |  |  |  |  |  |  |
| Weight | X |  |  |  | X |  |  |
| General condition | X | X | X | X | X | X | X |
| General blood test | X | X | X | X | X |  |  |
| Biochemical blood count a, b | X | X | X | X | X |  |  |
| Electrocardiogram | X |  |  |  |  |  |  |
| Review and assessment of adverse events |  | X | X | X | X | X | X |
| X-ray/multislice computed tomography | X |  |  |  | X |  |  |
| Polymerase chain reaction | X |  |  |  | X |  |  |
| Completion of individual study chart | X | X | X | X | X | X | X |
| a: glucose, calcium, sodium, potassium, carbon dioxide, chloride, albumin, amount of total protein in the blood, alkaline phosphatase (ALP), alanine aminotransferase (ALT), aspartate aminotransferase (AST), total blood bilirubin and its fractions, blood urea nitrogen, creatinine, creatine kinase, C-reactive protein, ferritin b: prothrombin time / activated partial thromboplastin time (APTT) / fibrinogen, D-dimer, lactate dehydrogenase (LDH), troponin, IL-6. | | | | | | | |

**2 INTRODUCTION**

**2.1 Rationale for the study**

The global coronavirus disease 2019 (COVID-19) pandemic poses an unprecedented challenge to public health, social and economic life [1, 2]. The etiological agent of COVID-19 is a new member of the Coronaviridae family, which is closely related to the severe acute respiratory syndrome coronavirus (SARS-CoV) and was named SARS-CoV-2 according to the taxonomy of viruses [3]. Coronaviruses (CoV) are enveloped RNA viruses with positive meaning (+ssRNA) [4]. Enveloped CoV invades host cells and initiates infection through the fusion of viral and cell membranes [5, 6]. Membrane fusion is provided by a large transmembrane glycoprotein S type I on the viral envelope and a related receptor on the surface of host cells [7, 8]. The arrangement of glycoprotein S on the surface not only allows it to carry out membrane fusion, but also makes it a direct target for host immune responses, making it the main.

Both host proteases and viral proteases play a key role in the processes of viral infection and replication in human cells. SARS-CoV-2 S proteins bind to angiotensin-converting enzyme 2 (ACE2) receptors in host cells to initiate cell entry. Host proteases, such as transmembrane serine 2 cell surface protease, facilitate the binding of the SARS-CoV-2 S-protein to the ACE2 receptor with high affinity and efficient fusion of the viral membrane with the host lipid membrane through S-protein priming [11]. Once SARS-CoV-2 enters the host cells, it uses the host cell's mechanism to translate the ORF into viral RNA. The SARS-CoV-2 RNA genome is approximately 30 thousand bases with 14 ORFs. ORF1a and ORF1b are translated into pp1a and pp1ab polyproteins, which are cleaved into 16 NSPs by two viral proteases incorporated into these two polyproteins [12].

3CLpro hydrolyzes at least 11 cleavage sites in polyproteins to form a number of NSPs, including itself (NSP5) and other important viral proteins such as RNA-dependent RNA polymerase (RdRp, NSP12) and helicase (NSP13) (Fig. 1) [13]. It recognizes the sequence of Leu-Gln ↓ (Ser, Ala, Gly) in most sites where ↓ represents the cleavage site. 3CLpro is a three-domain (domains I–III) cysteine protease that is highly conserved among coronaviruses. 3CLpro SARS-CoV-2 has 95% sequence identity and a very similar three-dimensional structure to 3CLpro SARS-CoV [14]. The 3CLpro coronavirus has a non-classical catalytic dyad Cys-His in the gap between domains I and II [15]. The process of proteolytic cleavage begins with the release of pp1a and pp1ab at its own N- and C-terminus [16], followed by maturation and formation of an active dimer.

Figure 1. Diagram of the SARS-CoV-2 genome. ORF1a and ORF1b, which occupy two-thirds of the 30kb RNA genome, are directly translated into 2 polyproteins, pp1a and pp1ab. Polyproteins are processed by 3CLpro and PLpro to produce 16 non-structural proteins. Other ORFs encode four structural proteins and nine additional SARS-CoV-2 factors. E, shell protein; M, membrane protein; N, nucleocapsid protein.

With the emergence of COVID-19, numerous efforts have focused on the reuse of drugs that were previously approved to treat other diseases, such as human immunodeficiency virus (HIV) and hepatitis C virus (HCV). HIV and HCV viral proteases mediate the cleavage of polyprotein precursors in different places, which leads to the formation of mature individual proteins [17]. Protease inhibitors act as peptidomimetics that compete for binding sites of the active substrate of proteases, thereby preventing the cleavage of viral polyproteins.

The combination of lopinavir with ritonavir, an approved protease inhibitor for the treatment of HIV, was one of the first to be recommended for clinical trials for the treatment of COVID-19. During the SARS outbreak in 2003, the combination of lopinavir in combination with ritonavir was shown to reduce overall mortality and intubation time [18, 19]. As a rule, lopinavir is rapidly metabolized in vitro, and ritonavir is usually prescribed concomitantly with other protease inhibitors, since ritonavir increases the level of other protease inhibitors in plasma and increases their half-life and bioavailability by inhibiting the main cytochrome P450 isoforms CYP3A4 and CYP2D6 [20]. Co-administration of lopinavir with ritonavir can increase the area under the plasma concentration-time curve by more than 100 times [21]. However, the combination of lopinavir and ritonavir has shown no clinical benefit in the treatment of patients with severe COVID-19 compared to standard therapy.

In addition to clinical trials, many preclinical studies have tested the efficacy of existing FDA-approved protease inhibitors against SARS-CoV-2. Lo et al. [23] found that simeprevir significantly reduces the viral load of SARS-CoV-2 in vitro by inhibiting 3CLpro, and also inhibits RdRp activity. It has also been found that simeprevir can modulate the host's immune response and can act synergistically with remdesivir to improve antiviral activity in vitro. In another study combining virtual screening and experimental testing, approved protease inhibitors such as boceprevir, telaprevir, and nelfinavir demonstrated different activities in inhibiting SARS-CoV-2 protease and viral replication [24]. Jan et al. [25] also found that boceprevir and nelfinavir mesylate can inhibit the activity of 3CLpro SARS-CoV-2. Although these approved protease inhibitors have demonstrated activity against SARS-CoV-2 in viter.

After elucidating the X-ray structure of non-ligand 3CLpro SARS-CoV-2, Zhang et al. [26] developed compound 13b with IC50 0.67 ± 0.18 μM against recombinant SARS-CoV-2 3CLpro. The pharmacokinetic characteristic of 13b revealed a pronounced tropism to the lungs when administered to mice by inhalation. In addition to the non-ligand structure, the SARS-CoV-2 3CLpro ligand complex has also been used as a model to aid in the discovery of new drugs. Through a combination of virtual and high-throughput screening (HTS), ebselen was identified that showed an IC50 value of 0.67 ± 0.09 μM in the enzymatic assay and a semi-maximum EC50 value of 4.67 ± 0.80 μM in the SARS-CoV-2 cell test. In another study of dipeptidyl compounds in combination with the 3CLpro coronavirus, Rathnayake et al. [27] developed a new series of dipeptidyls that showed varying degrees of activity against the 3CLpro coronavirus in enzyme assays. Two compounds have demonstrated antiviral effects against inf.

The crystallographic structure of the ligand-protease complex also contributed to the discovery of another 3CLpro inhibitor, PF-00835231 (metabolite PF-0730481), which is currently being tested as part of the COVID-19 clinical trials (ClinicalTrials.gov ID: NCT04535167). Initially, PF-00835231 was identified in studies aimed at detecting ketone-based SARS-CoV 3CLpro inhibitors using ligand-protease structures solved by X-ray crystallography [28]. Preclinical studies have shown that PF-00835231 is a potent antiviral agent against SARS-CoV-2 3CLpro with an inhibition constant (Ki) in the nanomolar range. Its successful preclinical safety profile and absorption, distribution, metabolism, and excretion properties have contributed to its clinical research [29].

Natural products and their derivatives have been used to treat various diseases since prehistoric times. A large number of plant products and their components have demonstrated promising inhibitory activity against viral infections in humans [30-33]. In 2002, during the SARS-CoV outbreak in China, several clinical research projects were initiated on the use of traditional Chinese medicine (TCM) in combination with Western medicine for the treatment of SARS-CoV infection [34]. After checking the clinical data, the WHO stated that the judicious use of TCM against SARS-CoV will help reduce the mortality rate compared to Western medicine alone. In addition, combination therapy can reduce the overall cost and highlights the importance of using herbal products for the treatment of SARS-CoV [34].

Against this background, the search for plant-based products with potential efficacy for inhibiting the 3CL protease of the coronavirus is relevant.

Polyphenols are secondary metabolites of plants with many health benefits, including strong antioxidant properties that protect against oxidative damage by free radicals and prevent chronic diseases. In light of the ongoing pandemic, researchers have studied the antiviral efficacy of polyphenols against COVID-19. Ghosh et al. found that the green tea polyphenols epigallocate (EGCG), epicatechingallate, and gallocatechin-3-gallate strongly interact with one or both of SARS-CoV-2's Mpro catalytic residues [37], and later demonstrated that six polyphenols from Broussonetia papyrifera also inhibit the catalytic activity of Mpro [38].

Clinical trials of several plant compounds against SARS-CoV-2-3CL Pro have shown the promise of obtaining drugs against SARS-CoV-2 of plant origin. Most recently, the 3CL protease inhibitor NLC-001, an herbal product administered orally as a dietary supplement, received approval from the US FDA. Currently, clinical trials of NLC-001 for the treatment of COVID-19 are ongoing in Israel, while the pharmaceutical company Todos Medical Ltd. is evaluating options for commercializing the drug around the world [35]. In another study, a natural product, baicalein (5,6,7-trihydroxyflavone)-flavone, originally isolated from the roots of Scutellaria baicalensis and Scutellaria lateriflora, was identified as an inhibitor of SARS-CoV-2-3CL pro. Clinical trials have shown that baicalein is well tolerated in the treatment of acute or chronic hepatitis in China (clinical trial registration number CTR20132944) [36]. In fact, clinical trials of several.

On the basis of polyphenols of sumac tannins Rhus coriaria, the drug Rutan has been developed, presented in two dosages - for children and adolescents in the form of tablets, 25 mg and for adults in the form of tablets, 100 mg.

In these studies, the structural features of polyphenols, antiviral activity in vitro and in vivo models in the experiment, preclinical and clinical studies of the drug were studied, which made it possible to recommend it for use in COVID-19 coronavirus infection in adults and children.

**2.2 Background**

For the past 15 years, scientists at the Institute of Bioorganic Chemistry have been conducting research in collaboration with Chinese scientists. China is one of the leading countries in terms of virological research, and for several years the screening of the biological activity of natural compounds isolated by scientists from the Institute of Bioorganic Chemistry has been carried out by Chinese scientists. In 2015, the substance and tablets "Rutan 0.025" No. 20 were registered by the Ministry of Health of the Republic of Uzbekistan and approved for medical use against influenza.

In connection with the pandemic and the defeat of the world's population by the SARS-CoV-2 virus, leading research centers in the United States, Great Britain, China and other countries, where appropriate conditions have been created for working with viruses and screening for antiviral activity has been established, are selecting promising biologically active compounds as potential antiviral drugs against SARS-CoV-19.

Based on the foregoing, in order to establish the effectiveness of the drug "Rutan 0.025", the Institute of Bioorganic Chemistry of the Academy of Sciences of the Republic of Uzbekistan, together with a number of research institutions, conducted additional studies justifying the feasibility of using this drug for the treatment of COVID-19 infected patients, in particular, at the beginning of 2020, together with scientists from the Shanghai Institute of Materia and Medica of the Chinese Academy of Sciences, which is one of the world's leaders in the screening of biologically active compounds, a series of studies was conducted on the in vitro antiviral effect of the drug "Rutan 0.025" against the SARS-COV-2 coronavirus and it was found that the drug "Rutan 0.025" effectively inhibits two vital enzyme systems of the SARS-COV virus:

1. It inhibits the activity of the key enzyme 3CL hydrolase of the SARS-Co-V-2 virus, and the degree of inhibition under the action of Rutan 0.1a was 78.3% (10 μM), in vitro, which indicates the possibility of suppressing the reproduction of the virus in vivo (analogues-oseltamivir, lopinavir, ritonavir).

Inhibits RNA-dependent RNA polymerase SARS-Co-V-2 (IC50 = 291 nM). Rutan 0.1 also exhibited anti-SARS-Co-V-2 infectious activity in VeroE6 cell culture with half the maximum effective concentration (EC50) of 5.84 μM. The EC50 value for Remdesivir is 2.0 μM. (analogues - Ribavirin, Avifavir, Favipiravir, Galidesivir). Thus, Rutan 0.025 has a dual antiviral effect against the SARS-COV2 virus: a) inhibits the activity of 3CL protease, b) inhibits RNA-dependent RNA polymerase, and can be an effective drug for its treatment.

Based on the above facts, and also that "Rutan 0.025" was successfully used in the treatment of influenza, in 2020 a preliminary clinical study was conducted at the clinic of the Research Institute of Virology, the 1-city clinic of Tashkent, Namangan, Samarkand regions, clinical trials of the drug were organized "Rutan 0.025" for a possible reduction in the duration of SARS-COV-2 viremia. It should be noted that the drug was prescribed according to the instructions for use in the first 2 days, 2 tablets of 25 mg once a day, in the next 2 days - 1 tablet 3 times a day, on the fifth day - 1 tablet 2 times a day. The duration of the course of treatment was 5 days. The study included 300 patients aged 18 to 70 years. According to the results obtained, the drug weakens the virus by 73.7%, starting from the 5th day of illness, compared with the control (57.1%).

In the process of discussing the prospects of using Rutan 0.1 against the SARS-CoV-2 virus, fears arose that it, being an immunostimulant and interferon inducer, could cause a "cytokine storm". The Institute of Bioorganic Chemistry, together with the Institute of Immunology of the Academy of Sciences of the Republic of Uzbekistan, conducted a series of studies to find out the possibility of initiating a "cytokine storm" under the influence of Rutan. Based on the results of the study, it was found that Rutan does not have a pronounced immunotropic and immunostimulant effect.

The study of the induction of the main interferons alpha (IFN-alpha) and gamma (IFN-gamma) under the action of Rutan in a group of people suffering from SARS-CoV2 showed that it has mild properties of an interferon inducer. This, in turn, proves that Rutan cannot be the cause of the "cytokine storm". (Report attached).

Recently, scientists from the University of Munich showed that the Nsp1 protein of the COVID-19 virus inhibits the protein synthesis system in the host cell. Stopping protein synthesis leads to the complete suppression of one of the body's main defense systems against infection. As a result, the innate immune response is no longer in effect, as an important signaling cascade stops working due to Nsp1. It is the disabling of primary immunity that is one of the possible reasons for the development of deadly conditions of patients, including a cytokine storm. (Matthias Thoms1 et al. Science. 2020)

During the post-registration period, the Institute of Bioorganic Chemistry of the Academy of Sciences of the Republic of Uzbekistan produced and sold more than 50,000 packages of Rutan tablets of 0.025 g No. 20 to medical and preventive institutions, the Ministry of Health and the pharmacy chain. In addition, in connection with the Covid-19 pandemic, 19.5 thousand packages of the drug were submitted to the Ministry of Health of the Republic of Uzbekistan for use in the treatment of patients infected with COVID-19 coronavirus as an antiviral agent. Over the past period, the developer (and manufacturer) - the Institute of Bioorganic Chemistry has not received a single complaint regarding non-compliance with the requirements of the ND quality, as well as unforeseen side effects and undesirable effects of the drug "Rutan 0.025" No. 20.

It should be especially noted that the great interest of the Chinese company "FE LLC "China - Uzbekistan Medicine Technical Park" in the future of the drug Rutan, recommended for use as a highly effective antiviral agent, in connection with which the drug was additionally registered in the Republic of Uzbekistan - (Registration Certificate DV / M-03116/05/20;) and also approved a regulatory document on quality control of the drug ND 42-Uz-10090 dated 05/18/2020.

Based on the foregoing, the results of preliminary clinical studies to study the effectiveness of the drug Rutan in the treatment of patients with COVID-19 are submitted for consideration in order to conduct additional clinical trials and, based on the results, make appropriate changes and additions to the instructions for use of the drug.

**2.3 Risk/Reward Assessment**

**2.3.1 Known potential risks**

Adverse reactions: allergic reactions may develop.

Contraindications: individual hypersensitivity.

Drug interactions: interaction with other drugs has not been studied.

Caution: The drug should be kept out of reach of children and should not be used after the expiration date.

Overdose: There are no data on overdose of the drug. [1,2,3].

**2.3.2 Known potential benefits**

The original anti-influenza drug, Rutan 0.025, developed by the Institute of Bioorganic Chemistry of the Academy of Sciences of the Republic of Uzbekistan. In collaboration with scientists of the National Research Center for Epidemiology and Microbiology named after Academician N.F.Gamaleya and FSBI "State Research Center Institute of Immunology" FMBA of Russia conducted a study of the antiviral activity of the drug "Rutan 0.025", isolated from the plant Sumach tannin Rhuscoriaria (Totum). It showed high antiviral activity against various strains of influenza virus and belongs to the class of ellagotannins.

Later, it was tested by Chinese scientists, resulting in a demonstration of reduced exposure to SARS CoV-2 coronavirus in vitro (a new coronavirus identified as the cause of COVID-19 coronavirus disease).

Studies of general toxicology of Rutan 0.025 showed that it belongs to the V class of non-toxic substances, has no cumulative properties, has no toxic effects on the parameters of peripheral blood, pathomorphology of kidneys, liver and internal organs in experimental animals at long-term use. Studies of specific toxicology showed that the drug has no local excitatory, allergic, mutagenic, immunotoxic, teratogenic and embryotoxic effects. There is a slight increase in total protein, increased diuresis and a slight increase in hemoglobin concentration, erythrocyte and reticulocyte count (7-20%). The drug in in vitro experiments also has a moderate antimicrobial effect against Gram-positive and Gram-negative bacteria. It was also found that "Rutan 0.025" exhibits antiradical activity against stable free-radical DFPH (1,1-diphenyl-2-picrylhydrazine) and prevents oxidation of lipids and proteins in biomembranes. High antioxidant activity of the drug was demonstrated on the model of lipid peroxidation induced by the enzyme Fe2+/ascorbate in mitochondria. This is due both to their ability to chelate various metal ions and directly interact with active oxygen species: O₂, OH-radicals and singlet oxygen. Consequently, "Rutan 0.1" can be effective as an anti-tumor, anti-inflammatory agent. [1,2,3].

**2.3.3 Assessment of potential risks and benefits**

Given that "Rutan 0.1" and Rutan 0.025 is the world's first plant-based antiviral drug against SARS-CoV-2, and simultaneously inhibits two key enzyme systems of the virus, the success rate of antiviral action is high. The drug does not have locally-exciting, allergic, mutagenic, immunotoxic, teratogenic and embryotoxic effects. According to the latest WHO data, to date, there is not a single drug agent active against SARS-COV2. Also, taking into account the results of preclinical and clinical trials, with information on the toxicity of the drug, the potential benefits prevail over the risks**.**

**3 TASKS AND ENDPOINTS**

| SUBSTANTIATION | ENDPOINTS | ENDPOINT TASKS |
| --- | --- | --- |
| The primary (main) goal is to test the safety, efficacy of the drug "Rutan 0.1" and "Rutan 0.025" against COVID-19. | The primary endpoint is the assessment of the safety of the drug "Rutan 0.1" and "Rutan 0.025" according to clinical and laboratory data (compared with a comparable group, the clinical manifestations of the disease should not worsen, a critical assessment of the appearance of new symptoms or complaints in the study subject and their relationship with taking drugs). Assessment by laboratory tests (pronounced deviations of the results of the tests of the subjects from the comparable group).  Establishment of efficacy - assessment of a clinically significant therapeutic effect (reducing the duration of the course of the disease, alleviating the clinical manifestations of the disease, in contrast to a comparable group). | This clinical trial tests the efficacy of Rutan 0.1 and Rutan 0.025, which has not previously been used in the treatment of COVID-19. Therefore, it is necessary to evaluate the safety and efficacy profile of this dosage in patients with COVID-19. |
| The secondary goal is the possibility of combining the study drug Rutan with the current COVID-19 therapy at the time of the study. | If a drug interaction is detected and a regular detection of adverse events (drug interactions), the study will be stopped. |  |

**4 RESEARCH DESIGN**

**4.1 Overall design**

**Adults**

This study is a randomized, open-label, controlled trial to evaluate the safety and efficacy of a novel therapeutic agent, Rutan 0.1, in hospitalized adult patients diagnosed with COVID-19. The study is a multicentre trial that will be conducted at approximately three centers in the city of Tashkent, Uzbekistan. The study will be a series of comparisons with two groups. The main group will receive the drug "Rutan 0.1" along with the current at the time of the study, recommended by the national treatment protocol. The control group will not be given the study drug. There will be continuous monitoring to stop the study due to futility, efficacy or safety. Because of the likelihood that the baseline standards for maintenance therapy may evolve/improve over time, safety and efficacy comparisons will adapt. The Independent Data and Safety Monitoring Panel will actively monitor interim data to make recommendations for early closure of the study or changes in study groups.

Randomization will be based on:

• Card system (the patient will be asked to choose one of two cards with the same drawings on the visible side, and after choosing, when turning over the cards, it will be determined which group the patient will be included in)

• The severity of the disease at enrollment:

o Mild to moderate illness: SpO2 >94% and respiratory rate <24 breaths/min without supplemental oxygen. During hospitalization, subjects will be assessed daily. The duration of observation is about three months. Discharged patients will be asked to attend study visits on days 30 and 90. All patients will undergo a series of tests for efficacy, safety and laboratory tests.

A clinical trial is being conducted during the inpatient/outpatient treatment of a patient with COVID-19.

Patients of the main group (400 patients) will be prescribed Rutan 0.1 tablets, along with the therapy recommended at the time of the study. The course of treatment will be - on the first day, 1 tablet 3 times a day, in the following days - 1 tablet 2 times a day. The duration of the course of treatment is 10 days, with good tolerability of the drug.

The comparison group will receive the current recommended treatment at the time of the study without the use of study drug.

**Children**

This study is a randomized, open-label, controlled trial to evaluate the safety and efficacy of a new therapeutic agent, Rutan 0.025, in hospitalized children diagnosed with COVID-19. The study is a single-center trial that will be conducted at a specialized clinic in Tashkent, Uzbekistan. The study will be a series of comparisons with two groups. The main group will receive Rutan 0.025 along with the up-to-date, nationally recommended treatment protocol at the time of the study. The control group will not be given the study drug. All 201 patients will have their nasal secretions analyzed for other ARIs, including: RC - respiratory syncytial virus, Adenovirus, Rhinovirus, Parainfluenza 1, 2, 3, 4, Influenza A, Influenza B, Bocavirus, MP lymphoma virus). Also, 60 patients from the ARI unit without COVID-19 will be analyzed for the incidence and nature of ARI - control group. There will be ongoing monitoring to discontinue the study because of futility, efficacy, or safety. Because of the likelihood that background standards of maintenance therapy may evolve/improve over time, comparisons of safety and efficacy will be adapted. An independent data and safety monitoring committee will actively monitor interim data to make recommendations for early study closure or changes in study groups.

**Randomization will be done by:**

- Card system (the patient's caregiver will be asked to select one of two cards, with identical pictures on the visible side, and once selected, flipping the cards will determine which group the patient will be enrolled in)

- Degree of severity of illness at enrollment:

**-** Disease of mild to moderate severity: SpO2> 94% and respiratory rate < 24 breaths/min without supplemental oxygen. Subjects will be evaluated daily during hospitalization. The duration of follow-up is approximately three months. Discharged patients will be asked to attend study visits on days 30 and 90. All patients will undergo a series of efficacy, safety and laboratory tests.

The clinical study is conducted during the inpatient/outpatient treatment of a patient with COVID-19.

Patients in the main group (210 patients) will be prescribed Rutan 0.025 tablets along with the recommended therapy at the time of the study. The course of treatment will be 1 tablet 3 times a day, on the following days - 1 tablet 2 times a day. The duration of the course of treatment is 10 days, with good tolerance of the drug.

The comparison group will receive the current recommended treatment at the time of the study without the study drug.

**4.2 Scientific rationale for the study design**

There is currently no specific antiviral therapy for coronavirus infections. Few treatment studies have been done because most human coronavirus strains cause self-limiting disease and treatment is supportive. After severe acute respiratory syndrome coronavirus (SARS) was identified in 2002 and caused a major global outbreak, interest in developing a specific therapeutic agent increased [44-64].

Patients with SARS-CoV-2 have been treated with corticosteroids, IFN type 1 drugs, convalescent plasma, ribavirin, lopinavir, or ritonavir, and, with the exception of ribavirin, many of these drugs have preclinical in vitro data supporting their effectiveness, as SARS outbreaks, new therapeutic agents targeting viral entry proteins, proteases, polymerases, and methyltransferases have been tested, but none have been effective in clinical trials [44,45,46].

This study uses an adaptive design that maximizes our effectiveness in identifying a safe and effective therapeutic agent for COVID-19 during the current outbreak. Randomization is necessary to establish the efficacy of the new therapeutic agent. Finally, collection of clinical and virologic data on included patients using a standardized timeline and data collection tools should provide valuable information about the clinical course and disease associated with COVID-19 in a diverse group of hospitalized adult patients.

**4.3 Dose Rationale**

An analysis of the latest data on the use of similar antiviral drugs (Remsidivir, Favipiravir, Lopinavir) shows that the dose of the drugs used against SARS-CoV-2 is 5-8 times higher than in the previous clinical study of the drug "Rutan 0.025". A low concentration of this drug did not show significant efficacy in adults. Therefore, it was decided to increase the dose of the drug four times. The form of the drug used for the study: tablets 100 mg. It is taken orally, on the first day, 1 tablet 3 times a day, in the following days - 1 tablet 2 times a day. The duration of the course of treatment is 10 days, with good tolerability of the drug. With the possibility of adjusting the dose. [1,2,3].

The low concentration of this drug has not been studied in children. Therefore, it was decided to use this dose of the drug in children for the efficacy study. Form of the drug used for the study: 0.025 mg tablets. It is taken orally, on the first day 1 tablet 2 times a day, on subsequent days - 1 tablet 2 times a day. Duration of the course of treatment - 10 days, with good tolerance of the drug. With the possibility of adjusting the dose.

**4.4 Determination of study completion**

A participant is considered to have completed the study if he/she has completed all phases of the study, including the last visit or last scheduled procedure specified in the Schedule of Measures (SMA), Section 1.3.

Study termination is defined as the completion of the last visit or procedure specified in the GM in the study**.**

**5 STUDY POPULATION**

**5.1 Inclusion criteria**

**Adults**

1. Providing a signed and dated informed consent form.

2. Declared willingness to comply with all study procedures and availability during the study.

3. Man or woman over the age of 18.

4. Patients with PCR-confirmed COVID-19 with mild/moderate disease. U07.1.

Patients are registered on the basis of the document of the Republic of Uzbekistan: Interim recommendations for the management of patients infected with COVID-19 (sixth version) dated August 04, 2020.

5. Ability to take oral medications and willingness to adhere to the regimen.

6. For women of childbearing potential: use of highly effective contraceptives for at least 1 month before screening and agree to use such a method during study participation and for an additional 4 weeks after the end.

7. For men of reproductive potential: use condoms or other methods to ensure effective contraception with a partner.

8. Agree to adhere to the lifestyle principles (see Section 5.3) throughout the study period.

**Children**

1. Submission of a signed and dated informed consent form by the caregiver.

2. The caregiver's stated willingness to comply with all study procedures and availability for the duration of the study.

3. Children 8 to 18 years of age.

4. Children with a confirmed PCR assay for COVID-19 with mild/moderate disease. U07.1.

Patients are processed based on the Republic of Uzbekistan document: Temporary Recommendations for the Management of Patients Infected with COVID-19 (sixth version) dated August 04, 2020.

5. Ability to take oral medications and willingness to comply with the regimen.

6. Consent to adhere to lifestyle principles (see Section 5.3) for the duration of the study.

**5.2 Exclusion Criteria**

**Adults**

An individual meeting any of the following criteria will be excluded from participation in this study:

1. Pregnancy or lactation.

2. Severe form of COVID-19 disease. U07.1.

3. Treatment with another investigational drug.

4. Individual intolerance to the drug.

5. The occurrence of any allergic reactions.

6. Weighting of the general state of health of the patient and the transition to a severe form of the disease.

**Children**

Children who meet any of the following criteria will be excluded from participation in this study:

1. severe COVID-19 disease. U07.1.

3. treatment with another investigational drug.

4. individual intolerance to the drug.

5. Occurrence of any allergic reactions.

6.Aggravation of the patient's general state of health and transition to a severe form of the disease.

**5.3 Lifestyle**

In this study, participants are asked to:

- Refrain from consuming oranges, grapefruit or grapefruit juice, pomelo, exotic citrus fruits, grapefruit hybrids, or fruit juices, until the beginning and last dose.

- Refrain from foods containing caffeine or xanthine (e.g., coffee, tea, cola drinks, and chocolate) for the duration of treatment.

- Refrain from exercise for two hours before each blood collection for clinical laboratory tests. Participants may participate in light recreational activities (e.g., watching TV, reading) during the study.

**5.4 Study Admission Strategies**

The study sample includes men and women of any race and ethnicity over the age of 18 in the amount of 400 people and 210 children of all races and ethnicities between the ages of 6 and 18.

Rate of accrual - as patients are admitted to inpatient or outpatient care, after screening and informed consent is signed by the caregiver.

Source of participants - inpatient/ambulatory.

Types of planned hiring strategies - not considered.

**6 THE THERAPEUTIC AGENT UNDER STUDY**

**6.1** **"RUTAN 0.1" and "RUTAN 0.025" and its application**

**6.1.1 Therapeutic agent description**

Pharmacotherapeutic group: antiviral agent against influenza A- H3N2 and H1N1, B-(B1) and (O3) strains as well as SARS-CoV-2 coronavirus. The mechanism of action is realized intracellularly. It has direct virulicidal action.

The mechanism of action of Rutan 0.025 is based on the inhibitory effect of two key enzyme systems of RNA viruses:

1. inhibits the enzyme 3 CL protease with which the virus forms all the functional proteins that perform the pathological actions of the virus and structural proteins. Protease inhibition does not produce functional and structural proteins, resulting in an immature and incapable of infecting virus.

2. inhibition of RNA-dependent RNA polymerase results in inhibition of viral multiplication.

Studies of general toxicology of Rutan 0,025 showed that it belongs to V class of nontoxic substances, has no cumulative properties, has no toxic effect on peripheral blood parameters, pathomorphology of kidneys, liver and internal organs in experimental animals at long-term use. Studies of specific toxicology showed that the drug has no local excitatory, allergic, mutagenic, immunotoxic, teratogenic and embryotoxic effects. There is a slight increase in total protein, increased diuresis and a slight increase in hemoglobin concentration, erythrocyte and reticulocyte count (7-20%). The drug in in vitro experiments also has a moderate antimicrobial effect against Gram-positive and Gram-negative bacteria. It was also found that Rutan 0.025 exhibited antiradical activity against stable free-radical DFPH (1,1-diphenyl-2-picrylhydrazine) and prevented oxidation of lipids and proteins in biomembranes. The high antioxidant activity of the drug was demonstrated on the model of lipid peroxidation induced by the enzyme Fe2+/ascorbate in mitochondria. This is due both to their ability to chelate various metal ions and directly interact with active oxygen species: O₂, OH-radicals and singlet oxygen. Consequently, "Rutan 0.025" can be effective as an antitumor, anti-inflammatory agent.

Pharmacokinetics: polyphenolic compounds on the basis of which the drug "Rutan 0.025" was created belong to the class of tannins. In the human body tannins break down into a number of metabolites that are difficult to investigate. Tannins in the amount of 3-6% only in the form of metabolites are determined in feces and urine. The bulk of ellagotannins, however, are converted to undetectable metabolites.

**6.1.2 Dosage and administration**

**"RUTAN 0.1" for adults**

It is taken orally, on an empty stomach, on the first day, 1 tablet 3 times a day, in the following days - 1 tablet 2 times a day. The duration of the course of treatment is 10 days, with good tolerability of the drug. With the possibility of adjusting the study dose of the drug.

**"RUTAN 0.025" for children**

Taken orally, on an empty stomach, 1 tablet 2 times a day. Duration of treatment course - 10 days, with good tolerance of the drug. With the possibility of adjusting the study dose of the drug.

**6.2 Preparation/processing/storage/reporting**

**6.2.1 Acquisition and accountability**

**Acquisition and Accountability**

Therapeutic agents will be delivered to the internal clinic pharmacy of the Virology Research Institute directly from the manufacturer or from the sponsor.

**Accountability:**

Researchers are responsible for the distribution and disposal of investigational products and are fully responsible for the accountability of investigational products. The internal pharmacist of the Virology Research Institute clinic pharmacy will be responsible for maintaining complete records and documentation of receipt of investigational product(s), reporting, dispensing, storage conditions and final disposition of the investigational product(s). The time the investigational product was given to the subject will be recorded on the appropriate data collection form. All investigational products, whether administered or not, must be documented in the appropriate record or dispensing log of the investigational product(s). The sponsor's monitoring staff will check the study product records and dispensing logs according to the monitoring plan.

**Disposition:**

After the end of the study treatment period or, as the case may be, during the course of the study after the study product record is completed, unused and used blisters of the active ingredient should be disposed of as indicated:

**Unused and used active ingredient blisters:**

o Must be returned to the sponsor or destroyed on site in accordance with applicable on-site procedures or the vendor's chosen disposal facility. Follow the facility's established hazardous materials destruction procedure or policy/standard operating procedure (SOP) for destruction of research products when destroying used and unused items.

**6.2.2 Formulation, Appearance, Packaging, and Labeling**

Active ingredient: "Rutan 0.1" and "Rutane 0.025" (3,6-bis-O-galloyl-1,2,4-tri-O-galloyl-β-D-glucose).

Excipients: potato starch, calcium stearate, lactulose.

Packing form: tablets 100 mg. 10 tablets in a planimetric package made of polyvinyl chloride film or imported lacquered printed aluminum foil. Two blister packs along with instructions for use, which are put into a box of cardboard boxes.

Packing form: tablets 25 mg. There are 10 tablets each in a polyvinyl chloride film or imported aluminum foil lacquered. Two per contour cell packing, together with instructions for use, which is placed in a box of cardboard.

**6.2.3 Storage and stability of the product**

Storage conditions: in a dry, dark place at a temperature not exceeding 25 ℃.

Shelf life: 2 years.

**6.2.4 Preparation**

Not required

**6.3 Error minimization measures: randomization and masking**

A parallel-group clinical trial model can be illustrated as follows:

Screening -- Inclusion -- Randomization -- Preparation period -- Baseline condition --

"Treatment a" + "Rutane 0.025" -- Outcomes at

"Treatment a" -- Outcomes x

The clinical study does not involve masking.

**6.4 Study Order**

Subjects will be evaluated by general clinical and biochemical blood tests; instrumental methods.

Documents required during the study: a sheet of informed voluntary consent of the patient (Appendix № 2), the card of an inpatient or/and outpatient, the journal of taking medication participants, the journal of the termination of participation / exit of the participant from the study.

**6.5 Accompanying Therapy**

All medications taken during treatment must be classified as over-the-counter and/or supplements. In other cases, however, medications are covered by a prescription from the treating physician.

**6.5.1 Medical Emergencies**

The research site will provide the necessary facilities for emergency, adverse events of the study drug.

Although the use of emergency medications is acceptable at any time during the study, their use should be delayed if possible. The date and time of administration as well as the name and dosing regimen of the remedies used should be recorded.

**7 STUDY TERMINATION AND TERMINATION/PARTICIPANT WITHDRAWAL**

**7.1 Termination of the study**

Termination of participation/ participant withdrawal means termination of participation in the study, and the remaining study procedures must be terminated and replaced with another participant as indicated in the study protocol. Any clinically significant changes in the patient's condition and/or laboratory values will be reported as an adverse event (AE) by the investigator. The investigator has the right to modify or terminate the study. Data to be collected at the time of study termination will include the following:

Assessment of the general condition of the investigator will be done through examination, clinical and laboratory and instrumental examinations.

**7.2 Discontinuance/withdrawal of participant from the study**

Participants can withdraw from the study at any time upon request.

The researcher may terminate participation or exclude a participant from the study for the following reasons:

- Significant noncompliance with the study regimen.

- If any clinical adverse event (AE), laboratory abnormality or other condition arises, or a situation in which continued participation in the study would not be in the best interest of the participant

- Progression of disease requiring discontinuation of the study.

- If the participant meets an exclusion criterion (newly developed or not previously recognized) that precludes further participation in the study

- Participant cannot receive the drug within 10 days.

The reason for terminating the participant's participation or withdrawing from the study will be recorded in the participant's withdrawal/exclusion log. Subjects who sign an informed consent form and are randomized but do not receive study participation may be replaced. Subjects who sign an informed consent form, are randomized and receive study participation, and then withdraw, or are excluded, or terminate study participation, will be replaced.

**7.3 Recall and re-visit issues**

A participant will be considered lost to follow-up if he or she fails to return for scheduled visits/recalls one and three months after completion of the study drug and is unable to contact research center staff.

If the participant is unable to return to the clinic or submit a recall, the following steps must be taken:

- The researcher will attempt to contact the participant and reschedule the missed visit for the next two weeks and advise the participant of the importance of following the assigned visit schedule and whether the participant is willing and/or required to continue participation in the study.

- Before a participant is deemed lost to follow-up, the researcher or designee will make every effort to re-establish contact with the participant (3 phone calls, if possible, and, if necessary, a registered letter to the participant's last known mailing address or local equivalent methods). These attempts at contact should be documented in the participant's medical record or study file.

- If the participant is still unavailable, he or she will be considered to have dropped out of the study for the primary reason of loss to follow-up.

**8 RESEARCH METHODS AND PROCEDURES**

**8.1 Performance Assessment**

Screening of patients admitted with COVID-19.

- Examination of the patient:

- Physical examination and vital signs

- Complete blood count with differential, with emphasis on trend of total lymphocyte count.

- Complete metabolic panel.

- Glucose.

- Calcium.

- Sodium, potassium, carbon dioxide, and chloride.

- Albumin

- Total protein

- Alkaline phosphatase, alanine transaminase (ALT) and aspartate aminotransferase (AST).

- Bilirubin

- Blood urea nitrogen and creatinine

- C-reactive protein (CRP)

- Ferritin

- D-dimer

- Computed tomography of the chest (if not done in the emergency room)

- Electrocardiography

See appendix #1.

Differentiation of disease severity COVID-19

**Mild form of the disease:**

Individuals who have any of the following symptoms, without signs of dyspnea, shortness of breath on exercise, or presence of signs on radiographs or CT scans: - fever up to 38°C; (or no fever), - sweating, - general weakness, - cough, - malaise, - runny nose or sore throat, - sore throat, nausea, - vomiting and/or diarrhea, - myalgia and body aches, - headache, - taste and smell disorders.

**Moderate form of the disease:**

The clinical signs of PNEVMONIA are: - fever, - cough, - difficulty and rapid breathing, - but no signs of severe pneumonia, including SpO2 ≥ 94% when breathing room air Diagnosis can be made based on clinical signs, but imaging techniques such as radiography, CT, or chest ultrasound may be helpful in making the diagnosis and identifying or excluding pulmonary complications.

**Severe form of the disease:**

The clinical symptoms of PNEUMONIA are: - fever, - cough, - difficulty and rapid breathing plus at least one of the following signs: - respiratory rate ≥30 breaths/min; - severe respiratory distress; - SpO2 < 90% when breathing room air Diagnosis can be made based on clinical signs, but imaging techniques such as radiography, CT or chest ultrasound may be helpful in making the diagnosis and identifying or excluding pulmonary complications.

After patients are selected by disease form, written consent is taken to participate in the clinical trial. Developed questionnaire-questionnaire is filled in. Randomization with inclusion into one of two study groups using baseline therapy or baseline therapy including Rutan 0.1 and Rutan 0,025 is carried out**.**

**Patient management procedure**

**Visit first**

**Initiate inpatient or outpatient treatment.**

Carry out initial examination. General clinical (detailed blood count) and biochemical tests (glucose, calcium, sodium, potassium, carbon dioxide, chloride, albumin, total blood protein count, alkaline phosphatase (ALP), alanine aminotransferase (ALT), aspartate aminotransferase (AST), total blood bilirubin and its fractions, blood urea nitrogen, creatinine, creatine kinase, C-reactive protein, ferritin, IL-6) blood. Instrumental methods of investigation (abdominal ultrasound, chest radiography, multislice computed tomography (MSCT), electrocardiogram (ECG). Draw up an individual card of the patient under study.

**Second visit**

Perform physical examination daily/every other day during inpatient/outpatient treatment. Repeat the examination if elevated (if applicable)

**Visit three**

Daily/ Every other day during inpatient/outpatient treatment Perform physical exam. Repeat examination if elevated or clinically deteriorating (if applicable).

**Visit Fourth**

On day 5 of inpatient/outpatient treatment, perform physical examination.

Perform repeat laboratory and biochemical tests. Polymerase chain reaction test for new-type coronavirus (COVID-19). Instrumental data (if necessary).

Fill out questionnaires or refer to Section 1.3 of the Schedule of Events.

**Visit Fifth**

One month after inpatient/outpatient treatment

Evaluate the examinee's condition through telephone communication/ordered letter/local equivalent methods.

Complete questionnaires or refer to Section 1.3, Schedule of Activities.

**Visit Sixth**

Three months after inpatient/outpatient treatment.

Evaluate the examinee's condition through telephone communication/ordered letter/local equivalent methods.

Complete questionnaires or refer to Section 1.3, Schedule of Measures.

**Final Evaluations**

Based on the results of studies during clinical use of Rutan 0.1 and 0.025

For participants who may terminate or withdraw early, it is important to identify the rationale at the last visit. (See details in Section 7, Termination of Study Participation and Study Termination / Withdrawal).

**8.1.1 NEW Assessment**

The NEW assessment has demonstrated the ability to differentiate patients at risk for adverse outcomes. (Smith, 2016). This assessment is based on 7 clinical parameters. The NEW Score is used as a measure of effectiveness. This should be assessed when first evaluating a particular study day. These parameters can be obtained from the hospital chart using the last measurement prior to the assessment. This is recorded for the day received. i.e., the 3rd day score is obtained on the 3rd day and recorded as the 3rd day.

| Physiological parameters | 3 | 2 | 1 | 0 | 1 | 2 | 3 |
| --- | --- | --- | --- | --- | --- | --- | --- |
| Respiration rate | ≤8 |  | 9-11 | 12-20 |  | 21-24 | ≥25 |
| _O2 saturation_ | ≤91 | 92-93 | 94-95 | ≥96 |  |  |  |
| Supplemental oxygen in any form |  | yes |  | no |  |  |  |
| Body temperature | ≤35,0 |  | 35,1-36,0 | 36,1-38,0 | 38,1-39,0 | ≥39,1 |  |
| Systolic pressure | ≤90 | 91-100 | 101-110 | 111-219 |  |  | ≥220 |
| Heart rate | ≤40 |  | 41-50 | 51-90 | 91-110 | 111-130 | ≥131 |
| Level of consciousness |  |  | А |  |  |  | Г,Б or БС |

Level of consciousness = alert (A), awakened only by voice (D) or pain (B) and not responding (BS).

**8.2 Study safety and other measures**

A list and description of the following procedures/assessments, if applicable:

- Physical examination (height and weight, adequacy, assessment of various body systems).

- Vital signs (temperature, pulse, respiration).

- Electrocardiograms (ECG): ECG will be read on site, in paper format.

- Chest X-rays/MSCT.

- Biological specimen collection and laboratory tests.

- PCR - nasopharyngeal swab for COVID-19 analysis.

- Assessment of adherence to the investigational intervention, or see "Adherence to the Investigational Intervention. Research Intervention Adherence, Section 6.4.

- Introduction of questionnaires or other instruments for patient-reported outcomes, such as a daily diary.

- Assessment of adverse events. Describe the conditions for tracking current NUIs/NUIs.

**8.3 Adverse and serious adverse events**

General toxicology studies of Rutan 0.025 and 0.1 A have shown that it belongs to class V non-toxic substances, has no cumulative properties, has no toxic effect on peripheral blood parameters, kidney, liver and internal organ pathomorphology in experimental animals during long-term use. Studies of specific toxicology showed that the drug has no local excitatory, allergic, mutagenic, immunotoxic, teratogenic and embryotoxic effects.

**Side effects:** development of allergic reactions is possible.

Contraindications: individual hypersensitivity.

Drug interactions: interaction with other drugs has not been studied.

**8.3.1 Definition of an adverse event**

An adverse event refers to any adverse health event related to the use of the intervention in humans, irrespective of whether it is considered to be related to the intervention.

**8.3.2 Definition of serious adverse events**

An adverse event (AE) or suspected adverse reaction is considered "serious" if, in the opinion of the investigator or sponsor, it results in any of the following: death, a life-threatening adverse event, hospitalization or extension of existing hospitalization, permanent or significant disability or significant impairment of ability to perform normal vital functions, or a congenital anomaly/congenital defect. Significant medical events that may not result in death, be life-threatening, or require hospitalization may be considered serious if, based on appropriate medical judgment, they could endanger the participant and may require medical or surgical intervention to prevent one of the outcomes listed in this definition. Examples of such medical events include allergic bronchospasm requiring intensive treatment in the emergency room or at home, blood dyscrasias or seizures that do not result in hospital admission, or the development of drug dependence or substance abuse.

**8.3.3 Classification of adverse event**

**8.3.3.1 Severity of Event**

For adverse events (AEs) not included in the protocol-defined evaluation system, the following guidelines will be used to describe severity.

- Mild - events require minimal or no treatment and do not interfere with the participant's daily activities.

- Moderate - events cause a small level of discomfort or concern about therapeutic measures. Moderate - events may cause some interference with activities.

- Severe - events disrupt the participant's normal daily activities and may require systemic drug therapy or other treatment. Severe events are usually potentially life-threatening or result in disability. Note that the term "serious" does not necessarily mean "serious**.**

**8.3.3.2 Attitude towards the study**

All adverse events (AEs) must be related to the study intervention, which is evaluated by a physician who examines and evaluates the participant based on the temporal relationship and his/her clinical judgment. The degree of confidence in the causal relationship will be assessed using the categories below. In clinical trials, the product under investigation should always be suspect.

- Related - NIH is known to occur with the investigational intervention, there is a reasonable probability that the investigational intervention caused the NIH, or there is a temporal relationship between the investigational intervention and the event. Reasonable possibility means that there is evidence to suggest a causal relationship between the intervention under investigation and the NIH.

- Not related means there is no reasonable possibility that the administration of the investigational intervention caused the event, there is no temporal relationship between the investigational intervention and the onset of the event, or an alternative etiology has been established.

OR

- Definitely related - there is clear evidence of a causal relationship and other possible contributing factors can be excluded. The clinical event, including the abnormality from the laboratory test, occurs in a probable temporal relationship to the study intervention and cannot be explained by a concomitant illness or other medications or chemicals. The response to study cancellation (failure) must be clinically reliable. The event must be pharmacologically or phenomenologically certain, using a satisfactory recall procedure if necessary.

- Likely related - there is reason to assume a causal relationship, and the influence of other factors is unlikely. A clinical event, including a deviation from the norm from a laboratory test, occurs within a reasonable time after the intervention under investigation, is unlikely to be related to a concomitant disease or other medication or chemical, and follows a clinically reasonable response to withdrawal (refusal of treatment). Recall information is not required to meet this definition.

- Potentially related - there is some evidence indicating a causal relationship (e.g., the event occurred within a reasonable period of time after taking the study medication). However, other factors may have contributed to the event (e.g., participant's clinical condition, other related events). Although AE can only be rated as "possibly related" shortly after detection, it can be labeled as requiring additional information and subsequently upgraded to "probably related" or "definitely related," as appropriate.

• An association with an unlikely – a clinical event, including an abnormal laboratory test result, whose temporal association with the conduct of the study intervention makes the causal relationship unlikely (e.g., the event did not occur within a reasonable time of the study intervention) and to which other drugs or chemicals or underlying disease provide a plausible explanation (e.g., the participant's clinical condition, other concomitant methods treatment).

• Unrelated – AEs are completely independent of the intervention being investigated, and/or there is evidence that the event is definitely related to another etiology. The clinician must document an alternative, definitive etiology.

**8.3.3.3 EXPECTATION**

[Expectation] will be responsible for determining whether an adverse event (AE) is expected or unexpected. An EOI will be considered unexpected if the nature, severity or frequency of the event does not match the risk information previously described for the intervention under study.

**8.3.4 Timing and frequency of event assessment and follow up**

The occurrence of an adverse event (AE) or serious adverse event (SAE) may attract the attention of research personnel during research visits and interviews with the study participant seeking care or after review by the study observer.

All NNIs, including local and systemic reactions that do not meet the NNI criteria, will be reported on the appropriate case report form. Information to be collected includes event description, time of onset, clinician assessment of severity, relationship to study product (assessed only by those trained and authorized to make the diagnosis), and time of resolution/stabilization of the event. All NJs occurring during the study must be appropriately documented regardless of relatedness. All NJs will be monitored until adequate resolution.

Any illness presents at the time the participant is screened will be treated as baseline and not reported as a NJ. However, if the study participant's condition deteriorates at any time during the study, it will be reported as a NS.

Changes in the severity of the NU will be documented, allowing an assessment of the duration of the event at each level of severity. NUIs characterized as recurrent require documentation of the onset and duration of each episode.

The researcher will record all reportable events with onset dates occurring any time after informed consent up to 7 (for non-serious AEs) or 30 days (for SAEs) after the last day of study participation. At each visit, the researcher should inquire about the occurrence of an AE/SAE since the last visit. Events will be monitored for outcome information prior to resolution or stabilization.

**8.3.5 Reporting Adverse Events**

The investigator must record and report non-serious adverse events to the sponsor according to the reporting schedule outlined in the protocol. It is important to understand that sponsors have additional responsibilities under regulations that are not described in this template and should be included in the appropriate documentation.8.3.6 Сообщение о серьезных неблагоприятных событиях

It is important to understand that sponsors have additional responsibilities under the rules that are not described in this template and should be included in the appropriate SOPs. The sponsor must report any suspected adverse reactions that are serious and unexpected. The sponsor should report an adverse event as a suspected adverse reaction only if there is evidence of a causal relationship between the drug and the adverse event, such as:

(A) A single event that is unusual and known to be closely related to drug exposure (e.g., angioedema, liver damage, Stevens-Johnson syndrome);

(B) One or more events that are not normally associated with drug exposure but are otherwise unusual in the population exposed to the drug (e.g., tendon rupture);

(C) A cumulative analysis of specific events observed in a clinical trial (such as known effects of an underlying disease or study condition, or other events that typically occur in the study population independent of drug therapy) that indicates that these events occur more frequently in the drug treatment group than in the concurrent or historical control group."

The clinical investigator immediately reports any serious adverse event to the sponsor, whether or not it is thought to be related to the study intervention, including those listed in the investigator's protocol or brochure, and should include an assessment of whether there is a reasonable possibility that the study intervention caused the event. Study endpoints that are serious adverse events (e.g., all-cause mortality) should be reported according to the protocol unless there is evidence indicating a causal relationship between the study intervention and the event (e.g., death from anaphylaxis). In this case, the researcher should immediately report the event to the sponsor.

All serious adverse events (SAEs) will be monitored until satisfactory resolution is resolved or until the investigator on site considers the event to be chronic or the participant is stable. Other supporting documentation of the event may be requested by the Data Coordination Center (DCC)/study sponsor and should be provided as soon as possible.

The study sponsor will be responsible for notifying of any unexpected fatal or life-threatening adverse reaction as soon as possible, but in no case later than 7 calendar days after the sponsor's initial receipt of the information. In addition, the sponsor must notify quality management and all participating investigators in the Investigational New Drug (IND) safety report of potential serious risks associated with the clinical trial or from any other source as soon as possible, but in no case later than 15 calendar days after the sponsor determines that the information is appropriate for reporting.

**8.3.7 Event Reporting to Participants**

Study participants will be informed of every adverse event and/or serious adverse event.

**8.4 Unanticipated Problems**

**8.4.1 Definition of Unanticipated Problems**

Unforeseen problems are considered problems involving risk to participants or others, including generally any incident, experience, or outcome that meets all of the following criteria:

- Unexpected in nature, severity, or frequency given (a) research procedures described in protocol-related documents;

- Related or possibly related to study participation ("possibly related" means that there is a reasonable possibility that the incident, experience, or outcome could have been caused by the procedures involved in the study); and

- Assumes that the research exposes participants or others to a greater risk of harm (including physical, psychological, economic, or social harm) than previously known or recognized.

**8.4.2 Communicating Unforeseen Problems to Participants**

Study participants will be informed of each unanticipated problem.

**9 STATISTICAL CONSIDERATIONS**

**9.1 Statistical Hypotheses**

The null hypothesis-the drug Rutan 0.1 for adults and 0.025 for children is unsafe and ineffective.

Alternative hypothesis- the drug is safe and effective.

If the drug is found to be unsafe, the dose of the drug is reduced or cancelled. No efficacy evaluation will be made.

**9.2 Determining Sample Size**

**Sample size**

The sample size is not calculated based on the statistical assumptions in the study. It is planned to enroll about 400 subjects and distribute them in a 1:1 ratio to basic therapy and Rutan.

**9.3 Populations for analysis**

Primary analyses will be based on the intention-to-treat population, including randomized participants. Similarly, safety analyses will be based on a modified intent-to-treat population consisting of all participants who received at least one infusion.

**9.4 Statistical Analysis**

The statistical analysis will test the hypothesis that the intervention reduces the rate of hospitalization and/or death relative to usual care. Decisions of superiority will be made separately for each intervention that enters the study

**9.4.1 General Approach**

This was identified by a suspicious randomized trial that results in a sense of redundancy with a type I bilateral external error rate of 0.05. Secondary suggestions are sorted by relative importance. They will be justified according to the selected summary statistics (eg proportions for categorical data, means with 95% confidence intervals for continuous data, median for time-to-event data). A planned statistical analysis will be developed.

The generally accepted stratified test for the initial assessment of the disease. The distribution of results by severity will be summarized by treatment group as a percentage. Fairness of coverage will be rated and verified.

Participants' baseline characteristics are summarized both overall and by randomized group, including stratification factors and important predictive and universal characteristics.

**9.4.2 Primary Endpoint Analysis**

A stratified hypothesis test will be used to determine baseline disease severity. The severity distribution of outcomes will be summed over the treatment group as a percentage. The fairness of the proportionality assumption will be evaluated and tested.

**9.4.3 Secondary endpoint analysis**

1) Differences in time-to-event endpoints (e.g., time to improvement by one category on an ordinal scale) by treatment will be summarized using Kaplan-Meier curves and 95% confidence limits.

2) Changes in ordinal scale at specific points in time will be summarized by proportion (e.g., proportion of those with 1-, 2-, 3-, or 4-point improvement or 1, 2, 3, 4-point deterioration)

3) Duration of event (e.g., duration of IVL) will be summarized according to median days with quartiles.

4) Morbidity data (e.g., frequency of new oxygen use) will be summarized as percentages with a 95% confidence interval.

5) Categorical data (e.g., 28-day mortality or ordinal scale by day) will be summarized according to proportions with confidence intervals of difference or odds ratios for binary or multiple categorical scales, respectively.

**9.4.4 Missing data**

Missing data will be defined in the protocol and analyzed under the assumption of missing at random. The primary analysis will be performed using only complete cases. If the missing data rate is > 5% when an intervention is complete, sensitivity analyses will be performed using multiple imputation.

**9.4.5 Safety analysis.**

Safety endpoints include death before day 28, SNF, discontinuation of investigational infusions, and severe NS. These events will be analyzed as single-factor and as a composite endpoint. Time-to-event methods will be used for death and the composite endpoint. Each NUI will be counted once for a given participant and evaluated for severity and association with COVID-19 or study intervention. NUIs will be coded using the current version of the Medical Regulatory Activity Dictionary (MedDRA). The NJs will be represented by systemic organ class, duration (in days), start date, and end date. Adverse events leading to premature termination of study participation and serious treatment-related NIH should be presented in a table or a list.

**9.4.6 Basic descriptive statistics**

Baseline characteristics will be summarized by treatment group. For continuous measures, the mean and standard deviation will be summarized. Categorical variables will be described by the proportion in each category (with corresponding sample size numbers).

**9.4.7 Planned Interim and Early Analysis**

Early analysis: an unannounced baseline endpoint assessment phase will be performed prior to determining the primary endpoint. Additional early analyses include monitoring of study inclusion, baseline characteristics, and follow-up rates throughout the study by the research team.

**9.4.8 Interim safety analysis**

Interim safety analyses will be performed on approximately 25%, 50%, and 75% of all participants. The safety analyses will assess serious NNIs by treatment group and test for differences using the Pocock cost function approach with a one-way Type I 0.025 error rate.

**9.4.9 Interim Performance Review**

The Lan-DeMets analog of the O'Brien-Fleming boundary cost function will be used to monitor the primary endpoint as a benchmark for the overall bilateral Type I error rate of 0.05. An intermediate efficiency analysis will be performed after the primary efficiency endpoint is selected at approximately 50%, 75%, and 100% of the total information.

Conditional power calculates the probability of obtaining a statistically significant outcome by the end of the trial given the data accumulated by then, including and assuming a hypothetical treatment effect (e.g., treatment effect assumed to determine sample size) after that. If the conditional power is less than 20% under the original trial assumptions, consideration should be given to discontinuing the trial. An independent statistical team will prepare these closed reports for review and recommendation. The analyses will be presented with hidden codes for treatment groups to protect against the possibility of the report falling into the wrong hands. An additional paper on statistical issues related to monitoring will be submitted prior to the interim analysis.

**10. ADDITIONAL DOCUMENTATION AND FORMS OF OPERATION**

**10.1 Regulatory, ethical aspects and rules for the supervision of the study**

This study will be conducted in accordance with the International Conference on Harmonization of Good Clinical Practice (ICH GCP), according to the requirements of the State Standard of the Republic of Uzbekistan on Good Clinical Practice under №O'zDSt 2765:2018, the Law of the Republic of Uzbekistan "On protection of citizens' health", the Regulations on conducting clinical research in the Research Institute of Virology of the Ministry of Health of RUz. The Ethical Committee of the Ministry of Health of the Republic of Uzbekistan will review and approve this protocol, related informed consent documents, recruitment materials, and study participant handouts or surveys prior to recruitment.

Any amendments to the protocol or consent materials will be approved by an ethics committee prior to implementation. Review and approval will occur at least annually throughout the study. The researcher will notify the ethics committee of deviations from the protocol.

The sponsor must obtain documentation of ethics committee approval for this protocol, informed consent documents, and related documents prior to participant recruitment, screening, and enrollment.

**10.1.1 Informed Consent Process. Requirements for parent/guardian permission and child consent (in the case of a minor)**

Informed consent is a process that begins before an individual agrees to participate in a trial and continues throughout their participation in the trial. Researchers or designated research staff will obtain the subject's informed consent as required by state and local regulations and policies.

Subjects' guardians will receive a brief and focused presentation of key information about the clinical trial verbally and with written consent. Key information about the study will be organized and presented in lay terminology and language, making it easier to understand why an individual may or may not want to participate.

Caregivers Participants in the study will be asked to read and review the consent form. Participants must sign a Voluntary Informed Consent Sheet (VIC) prior to any research procedures conducted specifically for this study. After signing, a copy of the VIC will be given to the study participant's guardian.

The informed consent document may be updated and participants will be re-consented to the requirements of the LDIS if necessary.

**10.1.1.2 Other Informed Consent Procedures**

Subjects' guardians will be asked to consent to additional blood collection, use of residual samples and samples for secondary testing. Additional blood will be drawn for secondary testing at each visit when blood samples are collected for testing.

Stored samples will be barcoded to maintain confidentiality. Studies with identifiable specimens and data may be conducted as needed, but confidentiality of subjects will be maintained as described in this protocol and with the approval of the LDIS.

Secondary research specimens may be used to understand SARS-CoV-2 infection, the immune response to this infection, and the effects of therapeutic agents on these factors.

Samples will not be sold for commercial gain. Although the results of any future study may be patentable or commercially profitable, participants will have no legal or financial interest in any commercial development as a result of any future research.

There is no direct benefit to participants from additional samples collected or from secondary research. No results of secondary studies will be entered into the participant's medical record. Incidental findings will not be shared with the participant, including incidental findings requiring medical action, unless required by law.

Participant's guardians may revoke permission to use specimens for secondary use at any time. They will need to contact the study center and the specimens will be removed from the study repository upon completion of that study and completion of documentation outlining the reason for revoking permission to reuse specimens.

**10.1.2 Study Termination and Closure**

Section 7, "Termination of Study Intervention" and "Termination / Exclusion of Subject" describes the temporary termination of a study.

This research may be prematurely terminated for sufficient reasonable cause, including but not limited to:

- Determination of unexpected, significant, or unacceptable risk to subjects.

- Results of interim analysis

- Lack of compliance with protocol requirements

- Data that are not sufficiently complete and/or cannot be evaluated

- Regulatory Authorities.

If the study was prematurely terminated, the investigator will immediately inform study participants and the ethics committee, if applicable. The sponsor will notify the regulatory authorities, if applicable.

**10.1.3 Confidentiality and secrecy**

Confidentiality of information is strictly respected by participating investigators, their staff members, the sponsor and their agents. This confidentiality extends to clinical information pertaining to subjects, results of biological samples and genetic tests, and all other information obtained during study participation. No identifiable information about study subjects will be shared with unauthorized third parties. Confidentiality will be maintained when study results are published or discussed at conferences.

The study supervisor, other authorized representatives of the sponsor, representatives and/or regulatory agencies may review all documents and records to be maintained by the investigator. The Clinical Research Center will permit access to such records.

All original records, including electronic data, will be stored in secure systems in accordance with organizational policy and government regulations.

All study data and study samples that leave the center (including any electronic data transfer) will be identified only by a coded number that is linked to the participant through a code key maintained by the clinical research center. Names or easily identifiable information will not be disclosed unless the ethics committee approves and agrees with the consent form or in accordance with required reporting laws.

**10.1.4 Secondary use of stored specimens and data**

Secondary human research is the reuse of identifiable data or identifiable biological samples that were collected as a result of some other "primary" or "initial" activity, such as the data and samples collected in this protocol. However, any use of a specimen or data for secondary research purposes will be presented in a separate protocol and will require separate ethics committee approval.

Each specimen will only be labeled with a barcode and a unique tracking number to protect the confidentiality of the subject. Secondary studies may be conducted with coded samples and data, but participant confidentiality will be maintained as described for this protocol.

The participant's decision can be changed at any time by notifying the physicians or nurse investigators in writing. If the subject subsequently changes his or her decision, the samples will be destroyed if the samples were not used for research or released for a specific research project.

**10.1.4.1 Sharing data for secondary studies**

Data from this study can be used for secondary research. All individual participant data collected in the study will be available after de-identification. The SAP and analysis code will also be available. This data will be available immediately after publication without an end date.

The researcher can request that data about individual study subjects be removed from the data repositories if a study participant withdraws or changes his or her consent. However, some data that has been disseminated for approved research purposes may not be retrieved.

**10.1.5 Key Roles and Leadership of the Study**

The study is sponsored by the Institute of Bioorganic Chemistry of the Academy of Sciences of Uzbekistan. Decisions related to the study will be made by the research team.

**10.1.6 Safety Oversight**

**10.1.6.1 Research Team Oversight**

The research team will review the NU data pools every 2 weeks to ensure that there are no significant numbers of unexpected NU's (NU's that are not consistent with the known COVID-19 flow). If there is a significant number of unanticipated NU's, it will be suggested that the safety data be reviewed at a special meeting.

**10.1.6.2 Safety Oversight Committee**

Safety oversight will be performed by the Ministry of Innovation and the Agency for Sanitary and Epidemiological Welfare which will oversee the safety of the participants. The members of the above entities will be separate and independent from the research personnel involved in this trial and shall have no scientific, financial, or other conflicts of interest related to this trial.

The supervisory team will conduct the following checks:

- Every four weeks.

- A special meeting if the protocol team raises any issues

- A final review meeting after final completion of clinical database collection to review aggregate open safety data for this trial.

**10.1.7 Clinical monitoring**

Clinical site monitoring is conducted to ensure the protection of trial subjects' rights and welfare and the accuracy, completeness and validity of submitted trial data. Clinical monitoring will also ensure that the conduct of the trial complies with currently approved protocols/amendments, ICH, GCP, and applicable regulatory and sponsor requirements. Clinical monitoring will also confirm that all critical study procedures are performed in accordance with specific instructions in the protocol.

This study will be monitored by the Agency for Disease Control and Prevention. Details of clinical site monitoring are documented in the Clinical Monitoring Plan (CMP). The protocol details who will monitor, the frequency at which monitoring will occur, the level of detail at which monitoring will be performed, and the distribution of monitoring reports. Monitoring visits will include review of regulatory files, records, medical and laboratory reports, records of retention of interventions during field investigations, training records, and protocol and GCP compliance. Site observers will have access to each participant participating in the study, the research staff, and all study documentation in accordance with the observation plan

**10.1.8 Data processing and record keeping**

**10.1.8.1 Data Collection and Management Responsibilities**

Data collection is the responsibility of the research staff at the participating clinical trial center under the control of the research center. Clinical trial data from the original documentation (including, but not limited to, NNDs/NNDs, concomitant medications, medical history, physical assessments, and clinical laboratory data) will be entered by the clinical trial site into a dedicated logbook. The data system includes encryption protection and internal quality checks, such as range checks, to identify data that appear inconsistent, incomplete, or inaccurate. NRs and related drugs will be coded according to the most current versions of MedDRA and WhoDrug, respectively.

**10.1.8.2 Retention of Research Records**

Research-related records, including the regulatory file, reporting records for the investigational product, consent forms, subject source documents, and electronic records, must be retained for 2 years after the date of approval of the marketing application for the investigational product for the indication for which it is being investigated; or, if no application is submitted or the application is not approved for that indication, until 2 years after termination of the investigation. However, these records should be retained for a longer period if required by local rules or regulations. No records will be destroyed without the written consent of the Department of Innovation and the Agency for Disease Control. Specimen retention consent forms attached to identifiable specimens will be retained as long as the specimens remain in an identifiable format and for a minimum of three years after use of the identifiable specimens in human studies without exception.

**10.1.8.3 Baseline Records**

Baseline data is all information in the original records (and certified copies of the original records) about clinical outcomes, observations, or other activities in a clinical trial that is necessary to reconstruct and evaluate the trial. Source data must be attributable, legible, up-to-date, original, accurate, and complete.

Requesting medical records from the primary treating physician is required.

**10.1.9 Deviations from Protocol**

Deviation from protocol is any non-compliance with the clinical trial protocol, any process that is noted in the protocol, or GCP requirements, or any critical study procedures with specific instructions in the supporting documents referenced in the protocol.

Non-compliance can be either on the part of the participant, the investigator, or the research center personnel. In the case of deviation(s) in the field, corrective actions must be developed and implemented immediately. All individual deviations from the protocol will be reflected in the records of the case study.

All deviations must be reported promptly to the Ministry of Innovation and the Agency for Sanitary and Epidemiological Welfare and to the Ethics Committee of the Ministry of Health of the Republic of Uzbekistan in accordance with the procedures for reporting deviations from the protocol. A completed copy of the protocol deviation form must be kept in the regulatory file and in the participant's documentation if the deviation is of a specific nature.

**10.1.10 Publication and Data Sharing Policy**

Upon completion of the study, the results of this study will be published in a scientific journal. Data will be available immediately after publication, with no end date, with data sharing at the sponsor's discretion.

**10.1.11 Publication**

Upon completion of the study, the lead research center is expected to publish the results of this study in a scientific journal. This study will comply with the Publication and Data Sharing Policy/Regulations.

**10.2 Abbreviations**

| ALT | Alanaminaminotransferase |
| --- | --- |
| AH RUz | Academy of Sciences of the Republic of Uzbekistan |
| ACE2 | Angiotensin-converting enzyme |
| AST | Aspartate aminotransferase |
| AST | Activated partial thromboplastin time |
| BVRS | Middle East respiratory syndrome |
| including | including |
| HIV | Human immunodeficiency virus |
| GNC | State Scientific Center |
| DFPG | 1,1-diphenyl-2-picrylhydrazine |
| EU | Effective concentration |
| kD | kiloDalton |
| PRC | People's Republic of China |
| CT | Computed tomography |
| LDH | Lactate dehydrogenase |
| μM | microMetre |
| MSCT | Multislice computed tomography |
| NII | Research Institute |
| nM | nanoMeter |
| NM | adverse event |
| OT-PCR | Polymerase chain reaction with reverse transcription |
| RA | Rheumatoid arthritis |
| RNA | Ribonucleic acid |
| RAS | Systemic lupus erythematosus |
| SNP | Serious adverse event |
| SOP | standard operating procedures |
| US | United States |
| SARS | Severe acute respiratory syndrome |
| FGBU | Federal State Budgetary Institution |
| ALP | Alkaline phosphatase |
| ECG | Electrocardiogram |
| MedDRA | Medical Dictionary of Regulatory Activity |

**11 REFERENCES**

1. Keni R, Alexander A, Nayak PG, Mudgal J, Nandakumar K. COVID-19: Emergence, Spread, Possible Treatments, and Global Burden. Front Public Health (2020) 8:216. doi: 10.3389/fpubh.2020.00216

2. Liang Y, Wang M-L, Chien C-S, Yarmishyn AA, Yang Y-P, Lai W-Y, et al. Highlight of Immune Pathogenic Response and Hematopathologic Effect in SARS-CoV, MERS-CoV, and SARS-Cov-2 Infection. Front Immunol (2020) 11:1022. doi: 10.3389/fimmu.2020.01022

3. Coronaviridae Study Group of the International Committee on Taxonomy of Viruses. The species severe acute respiratory syndrome-related coronavirus: classifying 2019-nCoV and naming it SARS-CoV-2. Nat Microbiol (2020) 5:536–44. doi: 10.1038/s41564-020-0695-z

4. Cui J, Li F, Shi ZL. Origin and evolution of pathogenic coronaviruses. Nat Rev Microbiol (2019) 17:181–92. doi: 10.1038/s41579-018-0118-9

5. Harrison SC. Viral membrane fusion. Virology (2015) 479-480:498–507. doi: 10.1016/j.virol.2015.03.043

6. Shang J, Wan Y, Luo C, Ye G, Geng Q, Auerbach A, et al. Cell entry mechanisms of SARS-CoV-2. Proc Natl Acad Sci U S A (2020) 117:11727–34. doi: 10.1073/pnas.2003138117

7. Tang T, Bidon M, Jaimes JA, Whittaker GR, Daniel S. Coronavirus membrane fusion mechanism offers a potential target for antiviral development. Antiviral Res (2020) 178:104792. doi: 10.1016/j.antiviral.2020.104792

8. Ou X, Liu Y, Lei X, Li P, Mi D, Ren L, et al. Characterization of spike glycoprotein of SARS-CoV-2 on virus entry and its immune cross-reactivity with SARS-CoV. Nat Commun (2020) 11:1620. doi: 10.1038/s41467-020-15562-9

9. Walls AC, Park YJ, Tortorici MA, Wall A, McGuire AT, Veesler D. Structure, Function, and Antigenicity of the SARS-CoV-2 Spike Glycoprotein. Cell (2020) 181:281–92.e6. doi: 10.1016/j.cell.2020.02.058

10. Amanat F, Krammer F. SARS-CoV-2 Vaccines: Status Report. Immunity (2020) 52:583–9. doi: 10.1016/j.immuni.2020.03.007

11. Shang J Wan Y Luo C Ye G Geng Q Auerbach A Li F (2020) Cell entry mechanisms of SARS-CoV-2. Proc Natl Acad Sci USA 117:11727–11734.

12. Gordon DE Jang GM Bouhaddou M Xu J Obernier K White KM O’Meara MJ Rezelj VV Guo JZ Swaney DL, et al. (2020) A SARS-CoV-2 protein interaction map reveals targets for drug repurposing. Nature 583:459–468

13. Alexander SPH Ball JK Tsoleridis T (2020) Coronavirus (CoV) proteins (version 2020.5) in the IUPHAR/BPS Guide to Pharmacology Database. IUPHAR/BPS Guide to Pharmacology CITE 2020

14. Zhang L Lin D Sun X Curth U Drosten C Sauerhering L Becker S Rox K Hilgenfeld R (2020) Crystal structure of SARS-CoV-2 main protease provides a basis for design of improved α-ketoamide inhibitors. Science 368:409–412

15. Dai W Zhang B Jiang X-M Su H Li J Zhao Y Xie X Jin Z Peng J Liu F, et al. (2020) Structure-based design of antiviral drug candidates targeting the SARS-CoV-2 main protease. Science 368:1331–1335

16. Lee J Worrall LJ Vuckovic M Rosell FI Gentile F Ton A-T Caveney NA Ban F Cherkasov A Paetzel M, et al. (2020) Crystallographic structure of wild-type SARS-CoV-2 main protease acyl-enzyme intermediate with physiological C-terminal autoprocessing site. Nat Commun 11:5877

17. Lee J Worrall LJ Vuckovic M Rosell FI Gentile F Ton A-T Caveney NA Ban F Cherkasov A Paetzel M, et al. (2020) Crystallographic structure of wild-type SARS-CoV-2 main protease acyl-enzyme intermediate with physiological C-terminal autoprocessing site. Nat Commun 11:5877.

18. Chan KS Lai ST Chu CM Tsui E Tam CY Wong MM Tse MW Que TL Peiris JS Sung J, et al. (2003) Treatment of severe acute respiratory syndrome with lopinavir/ritonavir: a multicentre retrospective matched cohort study. Hong Kong Med J 9:399-406.;

19. Yao T-T Qian J-D Zhu W-Y Wang Y Wang G-Q (2020) A systematic review of lopinavir therapy for SARS coronavirus and MERS coronavirus-A possible reference for coronavirus disease-19 treatment option. J Med Virol 92:556–563

20. Wensing AMJ van Maarseveen NM Nijhuis M (2010) Fifteen years of HIV protease inhibitors: raising the barrier to resistance. Antiviral Res 85:59–74.

21. Hurst M Faulds D (2000) Lopinavir. Drugs 60:1371–1379, discussion 1380–1381.

22. Cao B Wang Y Wen D Liu W Wang J Fan G Ruan L Song B Cai Y Wei M, et al. (2020) A trial of lopinavir-ritonavir in adults hospitalized with severe Covid-19. N Engl J Med 382:1787–1799.

23. Lo HS Hui KPY Lai H-M He X Khan KS Kaur S Huang J Li Z Chan AKN Cheung HH-Y, et al. (2021) Simeprevir potently suppresses sars-cov-2 replication and synergizes with remdesivir. ACS Cent Sci 7:792–802.

24. Pathak N Chen Y-T Hsu Y-C Hsu N-Y Kuo C-J Tsai HP Kang J-J Huang C-H Chang S-Y Chang Y-H, et al. (2021) Uncovering flexible active site conformations of SARS-CoV-2 3CL proteases through protease pharmacophore clusters and COVID-19 drug repurposing. ACS Nano 15:857–872.

25. Jan J-T Cheng TR Juang Y-P Ma H-H Wu Y-T Yang W-B Cheng C-W Chen X Chou T-H Shie J-J, et al. (2021) Identification of existing pharmaceuticals and herbal medicines as inhibitors of SARS-CoV-2 infection. Proc Natl Acad Sci USA 118:e2021579118.

26. Zhang L Lin D Sun X Curth U Drosten C Sauerhering L Becker S Rox K Hilgenfeld R (2020) Crystal structure of SARS-CoV-2 main protease provides a basis for design of improved α-ketoamide inhibitors. Science 368:409–412.

27. Rathnayake AD Zheng J Kim Y Perera KD Mackin S Meyerholz DK Kashipathy MM Battaile KP Lovell S Perlman S, et al. (2020) 3C-like protease inhibitors block coronavirus replication in vitro and improve survival in MERS-CoV-infected mice. Sci Transl Med 12:eabc5332.

28. Hoffman RL Kania RS Brothers MA Davies JF Ferre RA Gajiwala KS He M Hogan RJ Kozminski K Li LY, et al. (2020) Discovery of ketone-based covalent inhibitors of coronavirus 3CL proteases for the potential therapeutic treatment of COVID-19. J Med Chem 63:12725–12747.

29. Boras B Jones RM Anson BJ Arenson D Aschenbrenner L Bakowski MA Beutler N Binder J Chen E Eng H, et al. (2020) Discovery of a novel inhibitor of coronavirus 3CL protease as a clinical candidate for the potential treatment of COVID-19. bioRxiv:2020.2009.2012.293498.

30. Ganta K. K., Mandal A., Debnath S., Hazra B., Chaubey B. (2017). Anti-HCV activity from semi-purified methanolic root extracts ofValeriana wallichii. Phytother. Res. 31, 433–440. 10.1002/ptr.5765

31. Panda S. K., Padhi L., Leyssen P., Liu M., Neyts J., Luyten W. (2017). Antimicrobial, anthelmintic, and antiviral activity of plants traditionally used for treating infectious disease in the Similipal Biosphere Reserve, Odisha, India. Front. Pharmacol. 8, 1–15. 10.3389/fphar.2017.00658

32. Shen M.-x., Ma N., Li M.-k., Liu Y.-y., Chen T., Wei F., et al. (2019). Antiviral properties of R. Tanguticum nanoparticles on herpes simplex virus type I in Vitro and in Vivo . Front. Pharmacol. 10, 1–13. 10.3389/fphar.2019.00959

33. Bahramsoltani R., Rahimi R. (2020). An evaluation of traditional Persian medicine for the management of SARS-CoV-2. Front. Pharmacol. 11, 1–29. 10.3389/fphar.2020.571434

34. WHO (2004). Clinical trials on treatment using a combination of traditional Chinese medicine and Western medicine. Geneva, Switzerland: WHO.

35. Golodetz K. S. (2020). 3CL protease inhibitor NLC-001 added to COVID-19-focused joint venture between Todos medical and NLC pharma. Available at: https://www.globenewswire.com/news-release/2020/09/17/2095292/0/en/3CL-Protease-Inhibitor-NLC-001-Added-to-COVID-19-focused-Joint-Venture-Between-Todos-Medical-and-NLC-Pharma.html

36. Li, M., Shi, A., Pang, H., Xue, W., Li, Y., Cao, G., et al. (2014b). Safety, tolerability, and pharmacokinetics of a single ascending dose of baicalein chewable tablets in healthy subjects. J. Ethnopharmacol. 156, 210–215. doi:10.1016/j.jep.2014.08.031

37. R. Ghosh, A. Chakraborty, A. Biswas, S. Chowdhuri Evaluation of green tea polyphenols as novel corona virus (SARS CoV-2) main protease (Mpro) inhibitors–an in silico docking and molecular dynamics simulation study J Biomol Struct Dyn (2020), pp. 1-13, 10.1080/07391102.2020.1779818

38. R. Ghosh, A. Chakraborty, A. Biswas, S. Chowdhuri Identification of polyphenols from Broussonetia papyrifera as SARS CoV-2 main protease inhibitors using in silico docking and molecular dynamics simulation approaches J Biomol Struct Dyn (2020), 10.1080/07391102.2020.1802347

39. Jin, Z. M. et al. Structure of Mpro from SARS-CoV-2 and discovery of its inhibitors. Nature 582, 289–293 (2020).

40. Chen, J. et al. Structural basis for helicase-polymerase coupling in the SARS-CoV-2 replication-transcription complex. Cell 182, 1560–1573 (2020).

41. Alsamri, H.; Athamneh, K.; Pintus, G.; Eid, A.H.; Iratni, R. Pharmacological and Antioxidant Activities of Rhus coriaria L. (Sumac). Antioxidants 2021, 10, 73. https://doi.org/10.3390/antiox10010073

42. Jin Z., Du X., Xu Y. et al. (2020) Structure of Mpro from COVID-19 virus and discovery of its inhibitors (https://doi.org/10.1038/s41586-020-2223-y).

43. Jia-Tsrong Jan, Ting-Jen Rachel Cheng, Yu-Pu Juang et al. Identification of existing pharmaceuticals and herbal medicines as inhibitors of SARS-CoV-2 infection. PNAS, January 15, 2021, 118 (5) e2021579118 (https://doi.org/10.1073/pnas.2021579118)

 44. Chan KS, Lai ST, Chu CM, et al. “Treatment of severe acute respiratory syndrome with lopinavir/ritonavir: a multicentre retrospective matched cohort study.” Hong Kong Med J 2003;9:399–406.

45. Loutfy MR, Blatt LM, Siminovitch KA, et al. “Interferon alfacon-1 plus corticosteroids in severe acute respiratory syndrome: a preliminary study.” JAMA 2003;290:3222–3228.

46. Wong VW, Dai D, Wu AK, et al. “Treatment of severe acute respiratory syndrome with convalescent plasma.” Hong Kong Med J 2003;9:199–201.

47. Barnard DL, Hubbard VD, Burton J, et al. “Inhibition of severe acute respiratorysyndrome-associated coronavirus (SARSCoV) by calpain inhibitors and beta-D-N4-hydroxycytidine.” Antivir Chem Chemother 2004;15:15–22.

48. Chu CM, Cheng VC, Hung IF, et al. “Role of lopinavir/ritonavir in the treatment of SARS: initial virological and clinical findings.” Thorax 2004;59:252–256.

49. Cinatl J Jr, Michaelis M, Scholz M, et al. “Role of interferons in the treatment of severe acute respiratory syndrome.” Expert Opin Biol Ther 2004;4:827–836.

50. Haagmans BL, Kuiken T, Martina BE, et al. “Pegylated interferon-alpha protects type 1 pneumocytes against SARS coronavirus infection in macaques.” Nat Med 2004;10:290 293.

51. Hensley LE, Fritz LE, Jahrling PB, et al. “Interferon-beta 1a and SARS coronavirus replication.” Emerg Infect Dis 2004;10:317–319.

52. Stroher U, DiCaro A, Li Y, et al. “Severe acute respiratory syndrome-related coronavirus is inhibited by interferon-alpha.” J Infect Dis 2004;189:1164–1167.

53. Tan EL, Ooi EE, Lin CY, et al. “Inhibition of SARS coronavirus infection in vitro with clinically approved antiviral drugs.” Emerg Infect Dis 2004;10:581–586.

54. Yamamoto N, Yang R, Yoshinaka Y, et al. “HIV protease inhibitor nelfinavir inhibits replication of SARS-associated coronavirus.” Biochem Biophys Res Commun 2004;318:719–725.

55. Zheng B, He ML, Wong KL, et al. “Potent inhibition of SARS-associated coronavirus (SCOV) infection and replication by type I interferons (IFN-alpha/beta) but not by type II interferon (IFN-gamma).” J Interferon Cytokine Res 2004;24:388–390.

56. Cinatl J, Morgenstern B, Bauer G, et al. “Glycyrrhizin, an active component of liquorice roots, and replication of SARS-associated coronavirus.” Lancet 2003;361:2045–2046.

57. Mair‐Jenkins J, Saavedra‐Campos M, Baillie JK, et al. “The effectiveness of convalescent plasma and hyperimmune immunoglobulin for the treatment of severe acute respiratory infections of viral etiology: a systematic review and exploratory meta‐analysis.” J Infect Dis. 2015; 211( 1): 80‐ 90.

58. Public Health England. Treatment of MERS-CoV: information for clinicians. Clinical decision-making support for treatment of MERS-CoV patients. September 2015.

59. Chan, J.F., et al., “Broad-spectrum antivirals for the emerging Middle East respiratory syndrome coronavirus.” J Infect, 2013. 67(6): p. 606-16.

60. Cheng, K.W., et al. “Thiopurine analogs and mycophenolic acid synergistically inhibit the papain-like protease of Middle East respiratory syndrome coronavirus.” Antiviral Res, 2015. 115: p. 9-16.

61. Wang, Y., et al., “Coronavirus nsp10/nsp16 Methyltransferase Can Be Targeted by nsp10- Derived Peptide In Vitro and In Vivo To Reduce Replication and Pathogenesis.” J Virol, 2015. 89(16): p. 8416-27.

# 62. <https://www.who.int/ru/emergencies/diseases/novel-coronavirus-2019/advice-for-public/q-a-coronaviruses>.

# 63.<https://www.who.int/ru/emergencies/diseases/novel-coronavirus-2019/global-research-on-novel-coronavirus-2019-ncov/solidarity-clinical-trial-for-covid-19-treatments>

# 64. <https://mininnovation.uz/ru/news/2095>
