# Supplementary material for: Repurposing of Rutan showed effective treatment for COVID-19 disease
Source: Front Med (Lausanne). 2023 Nov 29;10:1310129. doi: 10.3389/fmed.2023.1310129 (PMC10716264; doi:10.3389/fmed.2023.1310129)
Supplement: Supplementary file 2 [file Table_1.DOCX]

Supplementary Material

# Supplement to: Salikhov SI, Abdurakhmonov IY, Oshchepkova YI., et al. Repurposing of Rutan showed effective treatment for COVID-19 disease

## Supplementary Figures

***
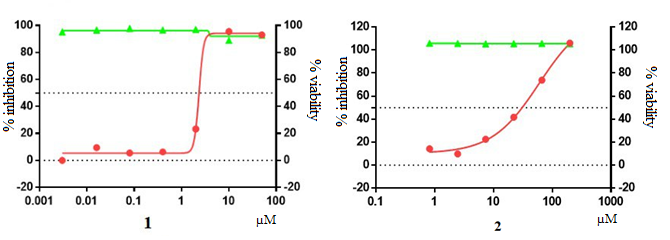
*Supplementary Figure 1.** Rutan - 1 and Favipiravir - 2 activity against H1N1 influenza virus in MDCK cells.

**
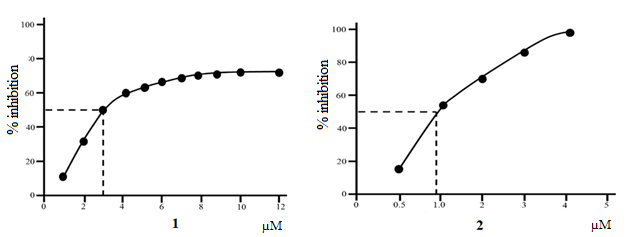
**

**Supplementary Figure 2.** Inhibition of SARS-CoV-2 3CL^pro^ by Rutan - 1 and Baicalein - 2

| 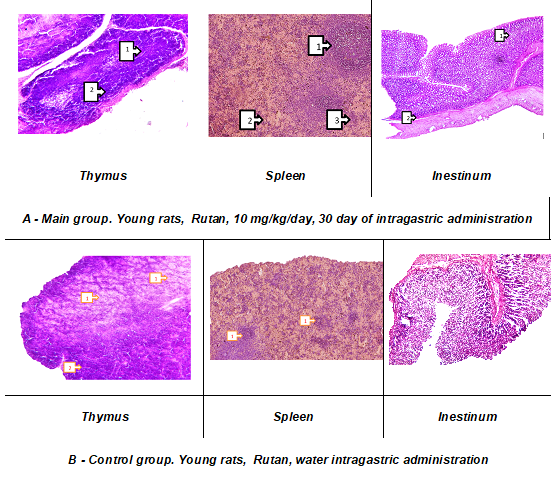  A – main group, Rutan, 10 mg/kg/day, 30 days of intragastric administration |
| --- |
| 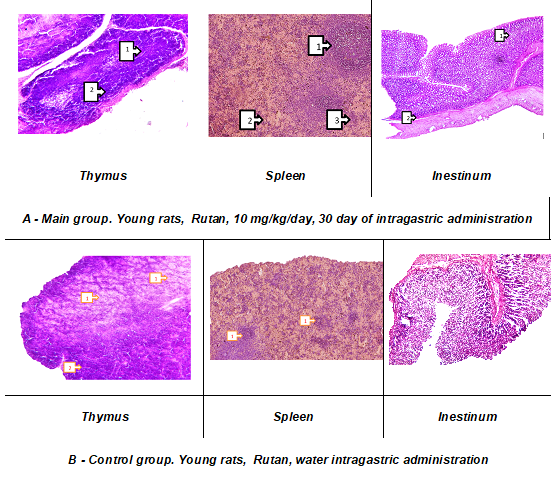  B – control group, water, 30 days of intragastric administration |
| **Supplementary Figure 3.** Histological picture of the immune organs after 30 days of intragastric administration of Rutan to developing (both sexes, aged 3-4 weeks old; weighing 25-30 g) Wistar Hannover rats. |


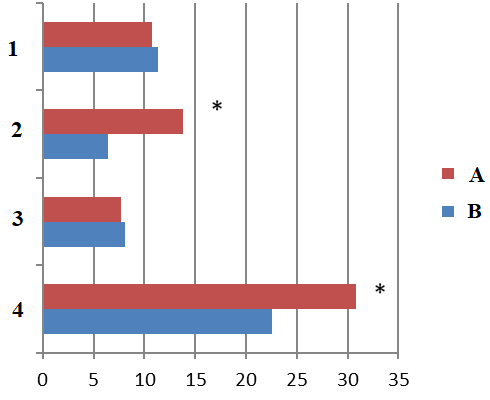


**Supplementary Figure 4. The frequency of detection of post-COVID syndromes** (1) - other manifestations, (2) - inflammatory-pain syndrome, (3) - catarrhal respiratory syndrome, (4) - astheno-vegetative syndromes in children aged 6-18 years of the main (В) (n=62) and control (А) (n=65) groups. Note: * - reliability of differences in indicators (P<0.05).

# Supplementary Tables

**Supplementary Table 1.** Main results of preclinical safety studies of Rutan substance conducted in adult animals

| **Test** | **Duration of experiment** | **Dose, type of administration, animals, group size** | **Death of animals/**  **Causes of death** | **Conclusion** |  |
| --- | --- | --- | --- | --- | --- |
| **General toxicology** | | | | | |
| **Single dose toxicity** | 14 days | 5000 mg/kg once, IG*, mice, 5 per group  2000, 5000 mg/kg once, IG*, rats, 5 per group  30, 50, 70, 80, 100 mg/kg, IV**, mice, 5 per group | 5 mice were used (4 mice were euthanized - the study design, one was found dead (toxicity)  10 rats (8 rats were euthanized: 6 - the study design, 2 - endpoint criteria***)  25 mice were used (24 mice were euthanized: 22 - the study design, 2 - endpoint criteria, one was found dead (toxicity) | Mice LD_50_ > 5000 mg/kg (Category 6 of toxicity);  rats LD_50_ = 4800 mg/kg (Category 5 of toxicity)  LD_50_ = 73 mg/kg  (Category 4 of toxicity) |  |
| **Cumulation** | 28 days | IG, mice, rats, 10 per group | 10 mice were used (10 were euthanized -the study design)   10 rats were used (10 were euthanized -the study design) | No cumulative properties |  |
| **Repeated dose toxicity** | 30 days | IG within 30 days:  25, 50, 100 mg/kg/day, rats, 5 per group;  10, 20, 40 mg/kg/day, rabbits, 3 per group | 20 rats were used (20 were euthanized - the study design)  12 rabbits were used (12 were euthanized - the study design) | No toxic effect on the condition of peripheral blood, kidney, and liver function, as well as specific changes in the internal organs of animals |  |
| **Specific toxicology** | | | | | |
| **Genotoxicity** |  | 0,5; 1,0; 2.0 mg; 4.0 mg/mL, culture medium |  | Absence of genotoxic properties (*in* *vitro* method of chromosomal aberrations in human peripheral blood lymphocytes) |  |
| **Reproductive and ontogenetic toxicity** | 20 days | 25, 100 mg/kg, IG, rats, 20 females and 10 males in groups | 180 rats:120 females (120 were euthanized- the study design), 60 males - no death | Absence of gonadotoxicity, embryotoxicity, or teratogenic effect |  |
| **Local tolerance** | | | | | |
| **Local irritant effect** | 10 days  14 days | 0.5 and 5% solution, intraconjuctivally once rabbits, 3 rabbits per group  0.5 and 5% solution, subcutaneously rats, 5 rats per group | 6 rabbits, no death  15 rats, no death | No local irritant effect  No local irritant effect |  |
| **Allergic properties** | 21 days  28 days | 25 mg/kg, IG,  guinea pigs 6 per group  0.5% and 5% solution cutaneously, guinea pigs  6 per group | 12 guinea pigs, no death   12 guinea pigs, no death | No allergic properties  No allergic properties |  |
| **Immunotoxicity** |  | 25, 50, and 100 mg/kg once, IG, mice 5 per group | 20 mice (20 were euthanized - the study design) | Immunomodulatory effect |  |
| **General pharmacology** | | | | | |
| **Effect on central nerve system** | 1 day | 50, 100 and 200 mg/kg, IG  Mice, 10 per group  50, 100 and 200 mg/kg, IG  Mice, 10 per group  50 mg/kg, IG  Mice, 5 per group  (2 series of experiments)  50 mg/kg, IG  Mice, 5 per group | 40 mice, no death  40 mice, no death  20 mice, no death  10 mice were used (10 were euthanized - endpoint criteria) | No effect on the orienting response  No effect on the spontaneous motor activity  No effect on the hypnotic activity of narcotic drugs  No anticonvulsant effect |  |

*IG – intragastric administration, **IV – intravenous administration

***** -** endpoint criteria for experiment animals were based on [21].

**Supplementary Table 2.** Main results of preclinical studies on the safety of the Rutan substance in developing rats

| **Test** | **Duration of experiment** | **Dose, type of administration, animals, group size** | **Death of animals/**  **Causes of death** | **Conclusion** |
| --- | --- | --- | --- | --- |
| **General toxicology** | | | | |
| **Acute Toxicity** | 14 days | 5000 mg/kg, once IG*;  5 per group  80, 100, 120, 160, 180 mg/kg, once IP**, developing rats, 5 per group | 5 developing rats (5 were euthanized - the study design)  25 developing rats (23 were euthanized: 17 - the study design, 6 - endpoint criteria***, and two were found dead (toxicity) | IG: LD_50_> 5000 mg/kg  (Category 6 of toxicity);  IP: LD_50_ = 137 (112÷165) mg/kg (Category 4 of toxicity) |
| **Cumulation** | 28 days | IG, developing rats, 10 per group | 10 developing rats were used (8 were euthanized - the study design), and two were found dead (cumulative toxicity) | No cumulative properties |
| **Repeated dose toxicity** | 30 days | 10, 20, and 40 mg/kg/day, IG for 30 days, developing rats, 5 per group | 20 developing rats were used (20 were euthanized - the study design) | No toxic effect on peripheral blood, kidney and liver function, or behavioral reactions. Histology: full-blooded arteries of parenchymal organs, hyperplasia of immune cells in primary and secondary immune organs |

* IG – intragastric administration, **IP – intraperitoneal administration

***** -** endpoint criteria for experiment animals were based on [21].

**Supplementary Table 3.** The frequency of several clinical symptoms in patients of the main and control groups upon admission to the hospital and before discharge with a mild course of the disease

| **Clinical symptoms** | **Main group (n=38)** | | | | **Control group (n=22)** | | | | **Chi^2^-test** |
| --- | --- | --- | --- | --- | --- | --- | --- | --- | --- |
|  | **Before treatment** | | **After treatment** | | **Before treatment** | | **After treatment** | | **р** |
|  | *n* | *%* | *n* | *%* | *n* | *%* | *n* | *%* |  |
| Weakness | 38 | 100 | 0 | 0 | 22 | 100 | 11 | 50 | <0,01 |
| Lethargy | 38 | 100 | 0 | 0 | 22 | 100 | 18 | 81 | <0,01 |
| Headache | 12 | 31.5 | 0 | 0 | 10 | 45.5 | 18 | 81 | <0,01 |
| Dizziness | 25 | 67.8 | 0 | 0 | 9 | 40.9 | 15 | 68.18 | <0,01 |
| Temperature | 18 | 47.36 | 0 | 0 | 12 | 54.5 | 13 | 59 | <0,01 |
| Chills | 34 | 89.5 | 0 | 0 | 14 | 63.6 | 0 | 0 | >0.05 |
| Irritability | 12 | 31.57 | 0 | 0 | 1 | 4.7 | 0 | 0 | >0.05 |
| Anxiety | 1 | 2.6 | 0 | 0 | 0 | 0 | 0 | 0 | >0.05 |
| Sweating | 12 | 31.57 | 0 | 0 | 1 | 4.7 | 0 | 0 | >0.05 |
| Itchy throat | 30 | 78.9 | 0 | 0 | 18 | 81.8 | 18 | 81 | <0,01 |
| Sore throat | 37 | 97.3 | 0 | 0 | 18 | 81.8 | 0 | 0 | >0.05 |
| Nasal congestion | 37 | 97.3 | 0 | 0 | 18 | 81.8 | 0 | 0 | >0.05 |
| Cough (dry, with phlegm) | 37 | 97.3 | 0 | 0 | 18 | 81.8 | 0 | 0 | >0.05 |
| Rhinitis | 14 | 36.8 | 0 | 0 | 9 | 40.9 | 0 | 0 | >0.05 |
| Dyspnea | 13 | 52 | 0 | 0 | 4 | 18.18 | 0 | 0 | >0.05 |
| Chest pain | 1 | 2.6 | 0 | 0 | 0 | 0 | 0 | 0 | >0.05 |
| Lack of air | 13 | 34.2 | 0 | 0 | 2 | 9 | 0 | 0 | >0.05 |
| Labored breathing | 2 | 5.5 | 0 | 0 | 0 | 0 | 0 | 0 | >0.05 |
| Anosmia | 37 | 97.3 | 0 | 0 | 20 | 52.63 | 10 | 45.45 | <0,01 |
| Ageusia | 38 | 100 | 0 | 0 | 20 | 52.63 | 10 | 45.45 | <0,01 |
| Anorexia | 0 | 0 | 0 | 0 | 0 | 0 | 0 | 0 | >0.05 |
| Dry mouth | 28 | 73.7 | 0 | 0 | 6 | 15.78 | 0 | 0 | >0.05 |
| Lower appetite | 35 | 92.1 | 0 | 0 | 21 | 95.45 | 0 | 0 | >0.05 |
| Nausea | 2 | 5.2 | 0 | 0 | 6 | 27.27 | 13 | 59 | <0,01 |
| Vomiting | 0 | 0 | 0 | 0 | 3 | 13.63 | 0 | 0 | >0.05 |
| Diarrhea | 0 | 0 | 0 | 0 | 0 | 0 | 0 | 0 | >0.05 |
| Myalgia | 13 | 34.2 | 0 | 0 | 5 | 22.72 | 6 | 27.27 | <0,01 |

**n**-number of samples

**Supplementary Table 4.** The frequency of several clinical symptoms in patients of the main and control groups upon admission to the hospital and before discharge with a moderate course of the disease with parenchymal damage up to 30%

| **Clinical symptoms** | **Main group (n=38)** | | | | **Control group (n=23)** | | | | **Χ²-test** |  |
| --- | --- | --- | --- | --- | --- | --- | --- | --- | --- | --- |
|  | **Before treatment** | | **After treatment** | | **Before treatment** | | **After treatment** | | **р** |  |
|  | *n* | *%* | *n* | *%* | *n* | *%* | *n* | *%* |  |  |
|  | 38 | 100 | 0 | 0 | 23 | 100 | 11 | 47.8 | <0.01 |  |
| Weakness | 38 | 100 | 0 | 0 | 23 | 100 | 19 | 82.6 | <0.01 |  |
| Lethargy | 35 | 92.1 | 0 | 0 | 8 | 34.78 | 19 | 82.6 | <0.01 |  |
| Headache | 18 | 47.4 | 0 | 0 | 11 | 47.8 | 15 | 65.21 | <0.01 |  |
| Dizziness | 33 | 86.8 | 0 | 0 | 15 | 65.2 | 15 | 65.21 | <0.01 |  |
| Temperature | 30 | 78.9 | 0 | 0 | 18 | 78.3 | 0 | 0 | >0.05 |  |
| Chills | 4 | 10.52 | 0 | 0 | 5 | 21.7 | 0 | 0 | >0.05 |  |
| Irritability | 1 | 97.3 | 0 | 0 | 0 | 0 | 0 | 0 | >0.05 |  |
| Anxiety | 22 | 57.89 | 0 | 0 | 12 | 52.2 | 18 | 78.2 | <0.01 |  |
| Sweating | 36 | 94.7 | 0 | 0 | 22 | 95.6 | 19 | 82.6 | <0.01 |  |
| Itchy throat | 36 | 94.7 | 0 | 0 | 22 | 95.6 | 0 | 0 | >0.05 |  |
| Sore throat | 34 | 89.5 | 0 | 0 | 18 | 78.3 | 0 | 0 | >0.05 |  |
| Nasal congestion | 34 | 89.5 | 0 | 0 | 17 | 73.9 | 0 | 0 | >0.05 |  |
| Cough (dry. with phlegm) | 20 | 52.63 | 0 | 0 | 12 | 52.2 | 0 | 0 | >0.05 |  |
| Rhinitis | 20 | 52.63 | 0 | 0 | 0 | 100 | 0 | 0 | >0.05 |  |
| Dyspnea | 16 | 42.1 | 0 | 0 | 0 | 100 | 0 | 0 | >0.05 |  |
| Chest pain | 20 | 89.5 | 0 | 0 | 5 | 21.7 | 0 | 0 | >0.05 |  |
| Lack of air | 10 | 26.3 | 0 | 0 | 5 | 21.7 | 0 | 0 | >0.05 |  |
| Labored breathing | 37 | 97.3 | 0 | 0 | 20 | 86.9 | 10 | 43.47 | <0.01 |  |
| Anosmia | 38 | 100 | 0 | 0 | 23 | 100 | 10 | 43.47 | <0.01 |  |
| Ageusia | 0 | 0 | 0 | 0 | 0 | 0 | 0 | 0 | >0.05 |  |
| Anorexia | 34 | 89.47 | 0 | 0 | 18 | 78.3 | 0 | 0 | >0.05 |  |
| Dry mouth | 32 | 84.2 | 0 | 0 | 23 | 100 | 0 | 0 | >0.05 |  |
| Low appetite | 5 | 13.2 | 0 | 0 | 15 | 65.2 | 13 | 56.52 | <0.01 |  |
| Nausea | 0 | 0 | 0 | 0 | 0 | 0 | 0 | 0 | >0.05 |  |
| Vomiting | 0 | 0 | 0 | 0 | 0 | 0 | 0 | 0 | >0.05 |  |
| Diarrhea | 25 | 65.78 | 0 | 0 | 11 | 47.8 | 6 | 26 | <0.01 |  |

**n**-number of samples

**Supplementary Table 5.** Dynamics of the virus content in the nasal secretion in patients of the main and control groups with a mild course of the disease

| **Virological study** | **Main group (n=38)** | | | **Chi2-test** | **Control group (n=22)** | | | **Χ²-test** |
| --- | --- | --- | --- | --- | --- | --- | --- | --- |
|  | **On admission** | **(+) After 5 days** | **(+) At discharge** | **p** | **On admission** | **(+) After 5 days** | **(+) At discharge** | **p** |
| PCR test for COVID-19 | 38  (100%) | 8  (21%) | 0 | <0,001 | 22  (100%) | 19  (86.4%) | 12  (54.5%) | <0.001 |

**Supplementary Table 6.** Dynamics of the virus content in the nasal secretion in patients of the main and control groups with a moderate course of the disease with parenchymal damage up to 30%

| **Virological study** | **Main group (n=38)** | | | **Chi2-test** | **Control group (n=23)** | | | **Χ²-test** |
| --- | --- | --- | --- | --- | --- | --- | --- | --- |
|  | On admission | (+) After 5 days | (+) At discharge | **p** | On admission | (+) After 5 days | (+) At discharge | **p** |
| PCR test for COVID-19 | 38  (100%) | 6  (15.8%) | 0  (0%) | <0,001 | 23  (100%) | 21  (91%) | 9  (39.2%) | <0.001 |

**Supplementary Table 7.** The frequency of several clinical symptoms in children aged 6-18 years of the main and control groups upon admission to the hospital and before discharge

| **Clinical symptoms** | **Main group (n=101)** | | | | **Control group (n=100)** | | | |  |
| --- | --- | --- | --- | --- | --- | --- | --- | --- | --- |
|  | **Before treatment** | | **After treatment** | | **Before treatment** | | **After treatment** | | **Χ²-test** |
|  | ***n*** | ***%*** | ***n*** | ***%*** | ***n*** | ***%*** | ***n*** | ***%*** | **p** |
|  | 101 | 100.0 | 101 | 100.0 | 100 | 100.0 | 100 | 100.0 | > 0.05 |
| Weakness, lethargy | 101 | 100.0 | 91 | 90.2 | 100 | 100.0 | 96 | 96.0 | > 0.05 |
| Malaise | 101 | 100.0 | 15 | 14.8 | 100 | 100.0 | 22 | 22.0 | > 0.05 |
| Hyperthermia | 98 | 97.0 | 5 | 4.9 | 95 | 95.0 | 22 | 22.0 | < 0.001 |
| Headache | 89 | 88.1 | 2 | 2.0 | 76 | 76.0 | 8 | 8.0 | < 0.05 |
| Pronounced sweating | 101 | 100.0 | 44 | 43.6 | 100 | 100.0 | 45 | 45.0 | > 0.05 |
| Dizziness | 39 | 38.6 | 15 | 14.8 | 44 | 44.0 | 18 | 18.0 | > 0.05 |
| Pharynx, redness, grainy | 81 | 80.2 | 29 | 28.7 | 90 | 90.0 | 42 | 42.0 | > 0.05 |
| Pain when swallowing | 69 | 68.3 | 5 | 4.9 | 68 | 68.0 | 17 | 17.0 | < 0,01 |
| Dry/ cough wet | 101 | 100.0 | 95 | 94.1 | 100 | 100.0 | 95 | 95.0 | > 0.05 |
| Pain with deep inhalation/exhalation | 42 | 15.0 | 0 | 0 | 15 | 15.0 | 8 | 8.0 | < 0.001 |
| Pain in muscles and bones | 89 | 88.1 | 2 | 2.0 | 83 | 83.0 | 1 | 1.0 | > 0.05 |
| Pain in the right hypochondrium | 5 | 4.9 | 0 | 0 | 5 | 5.0 | 0 | 0 | > 0.05 |
| Loss of taste and smell | 17 | 6.8 | 33 | 32.7 | 2 | 2.0 | 33 | 33.0 | < 0.01 |
| Loss of appetite | 91 | 90.1 | 8 | 7.9 | 83 | 83.0 | 7 | 7.0 | > 0.05 |
| Nausea, vomiting | 15 | 14.8 | 0 | 0 | 12 | 12.0 | 2 | 2.0 | > 0.05 |
| Diarrhea | 12 | 11.9 | 0 | 0 | 2 | 2.0 | 0 | 0 | > 0.05 |

**n**-number of samples

**Supplementary Table 8.** Distribution of patients in the catamnestic study by age group**.**

| **Groups/ age in years** | **6-10** | **11-14** | **15-18** | **Total** |
| --- | --- | --- | --- | --- |
| Control Count  Row % | 28  43.08 | 17  26.15 | 20  30.77 | 65 |
| Main Count  Row % | 18  29.03 | 25  40.32 | 19  30.65 | 62 |
| Total | 46 | 42 | 39 | 127 |
